# Exploring the risk of heat stress in high school pre-season sports training, Johannesburg, South Africa

**DOI:** 10.1007/s00484-024-02748-9

**Published:** 2024-08-14

**Authors:** Kayleigh Raines, Jennifer M. Fitchett

**Affiliations:** School of Geography, Archaeology and Environmental Studies, University of the Witwatersrand, Private Bag X3, Wits, 2050 South Africa

**Keywords:** Exertional heat stress, School sport, Thermal comfort indices, Outdoor thermal comfort, Pre-season training, Artificial surfaces

## Abstract

There is growing concern over the increased risk of heat stress induced by the changing climate, with increased global temperatures expected to exacerbate conditions. Heat stress in school sports is a critically understudied research area in South Africa (SA). As demonstrated by several studies conducted in the global north, the stress elicited by meteorological conditions on students participating in physical education lessons and after-school sports activities could have serious consequences on their health. This paper represents the first research in the region to investigate the risk of heat stress to school students during physical activity. Meteorological data were collected at a school in Johannesburg between March and July, which encompasses the seasons of late summer, autumn, and winter. These were inputted into four heat stress indices: Humidex, Heat Index, Wet Bulb Globe Temperature, and the Universal Thermal Climate Index, together with the measured Wet Bulb Temperature to determine the level of risk of heat stress during physical education lessons and after-school sports activities between March and July. Of the 2700 index scores calculated over this period, 56% indicate some level of heat stress, with almost 6% indicating a high and very high level of risk. Heat stress is calculated to be most likely to occur between 11h00-15h00, and the danger of pre-season training, which takes place in the summer months, is demonstrated by these results. Additionally, this research finds that heat stress is far more likely on artificial surfaces than on natural grass. The findings of this research intimate that school stakeholders should consider the implications of heat stress when considering timetabling and policy-making in the interests of safeguarding their students.

**Supplementary Information:**

The online version contains supplementary material available at 10.1007/s00484-024-02748-9.

## Introduction

Thermal comfort is a foundational concept within biometeorology, particularly within the scope of temperature-related health (Gosling et al. 2014). Thermal comfort arises from a complex interaction between the human body and its environment, wherein the body seeks to compensate for both its own heat production and loss, as well as external factors (Jendritzky and de Dear 2009; Gosling et al. 2014). This delicate state of balance becomes even more complicated when a human undertakes physical activity, where a greater interplay of physics, physiology, and behaviour exists (Brotherhood 2008). There is a narrow environmental range for thermal comfort, outside of which, behaviour changes in terms of level of activity and clothing choice are necessitated (Havenith 2002). Heat stress arises when a person is unable to compensate for the rise in temperature through physiological mechanisms (Hosokawa et al. 2021). Whilst heat stress can occur in the absence of physical activity – termed ‘classic heat stress’, exertional heat stress arises when the body is unable to compensate for increased core temperatures whilst engaging in physical activity (Garcia et al. 2022). Prolonged exposure to heat stress-inducing conditions whilst exercising could lead to exertional heat illness (EHI), particularly when vigorous exercise is undertaken, as this increases metabolic heat production (Hosokawa et al. 2021). Exertional heat stress occurs in many climatic regions around the world, where it can affect athletes participating in all sporting codes (Armstrong et al. 2007). The risks of exertional heat stress are heightened in persons who are unfit, unacclimatised, and in young (children) and old (elderly) populations (Périard et al. 2021). Schools take on considerable responsibility for the safety and wellbeing of their students, and thus understanding the heat risks in school sports is critical (Perry and McWilliam 2007).

This study is the first to explore experiences of thermal stress in the context of school sport in South Africa, with a particular focus on pre-season training. This is conducted through a series of on-site meteorological measurements taken during the time of school physical education (PE) lessons and sports training sessions, from which five thermal stress indices are computed.

## Literature review

Much of the existing research on EHI and heat stress in school- and college-aged sport has been conducted in the northern hemisphere, particularly in the USA (Hosokawa et al. 2021) and Canada (Vanos et al. 2016). Many northern hemisphere schools take extended breaks over the summer season, meaning that there is minimal school sporting activity during the warmer periods. Conversely, South African schools are in session throughout much of the warmest time of the year, and thus a great deal of both in-season and pre-season sports training takes place during this time. In addition, as a developing region, Africa is largely considered to be far more vulnerable to the effects of climate change than more developed regions (Ziervogel et al. 2014; Engelbrecht et al. 2015; Parkes et al. 2019). And whilst South Africa is generally considered to have the best scientific base for climate change studies on the continent, (Ziervogel et al. 2014), Roffe et al. (2023) point out that this research tends to focus on the effects that climate change will have on rainfall patterns, drought and flooding events, and tropical cyclones. Both Sherwood and Huber (2010), and Roffe et al. (2023) note the general lack of emphasis of research on the effect that climate change will have on extreme temperature events. Sports-related EHI is one of the leading causes of mortality in school- and college-aged athletes (Hosokawa et al. 2021), and accounts for more than 100 hospitalisations in the USA each year, (Gamage et al. 2020). It is possible that EHI is a currently under-reported cause of hospitalisation or death in South Africa. Heat stress is a relatively under-studied topic in the country, and there is no published research on heat stress in children during school physical activity in South Africa. Much of the research that does exist focusses on thermal comfort in the home, and the design of buildings to improve thermal comfort (e.g., Adesina et al. 2020; Wright et al. 2022). There is some research on thermal comfort in South African classrooms, but this is mostly limited to the work conducted by Gibberd and Motsatsi (2013), Bidassey-Manilal et al. (2016, 2020) and Pule et al. (2021).

Schoolchildren and students spend a significant proportion of their time indoors given the format of traditional education (de Dear et al. 2015; Pule et al. 2021). There is clear evidence that extreme temperatures have a negative effect on students, both inside the classroom and outdoors (Haverinen-Shaughnessy and Shaughnessy 2015; Wargocki et al. 2019; Shortridge et al. 2022). In an extensive study conducted in schools across Australia, de Dear et al. (2015) established that temperatures that exceed thermal comfort levels influence students’ concentration, memory, and efficiency. In a 1-year study conducted on grade 3 learners at Eastern Cape (SA) schools, Pule et al. (2021) found that both excessively low and excessively high ambient temperatures result in higher rates of absenteeism in schools. In terms of the geographical distribution of studies on classroom thermal comfort, the majority of studies are performed in Asia, Australia and Europe (Zomorodian et al. 2016; Pule et al. 2021; Lala and Hagishima 2022). There is a notable lack of literature pertaining to thermal comfort and heat stress in schools in Africa, both indoors and outdoors.

## Methods

The methodology used for this research is informed by the work of Hosokawa et al. (2021), where specific recommendations are made with regards to taking meteorological measurements. The use and interpretation of wet bulb globe temperature (WBGT) results is directed by the extensive work carried out by Yeargin et al. (2023). Guidance is taken from the work of Vecellio et al. (2022) in interpreting wet-bulb temperature (T_wb_) values, whilst Rothfusz (1990) informs the use of heat index (HI). Diaconescu et al. (2023) typify the use of humidex (Hx), and the work of Bröde et al. (2012) is consulted in the use of the universal thermal climate index (UTCI). Guidance is taken from the work of van der Walt and Fitchett (2021), and Roffe et al. (2023) when considering the implications of heat stress and outdoor thermal comfort in South Africa specifically. The study was approved for an institutional ethics waiver, and permission was obtained from St Andrew’s School to conduct the study on their grounds.

### Study site

**Fig. 1:**
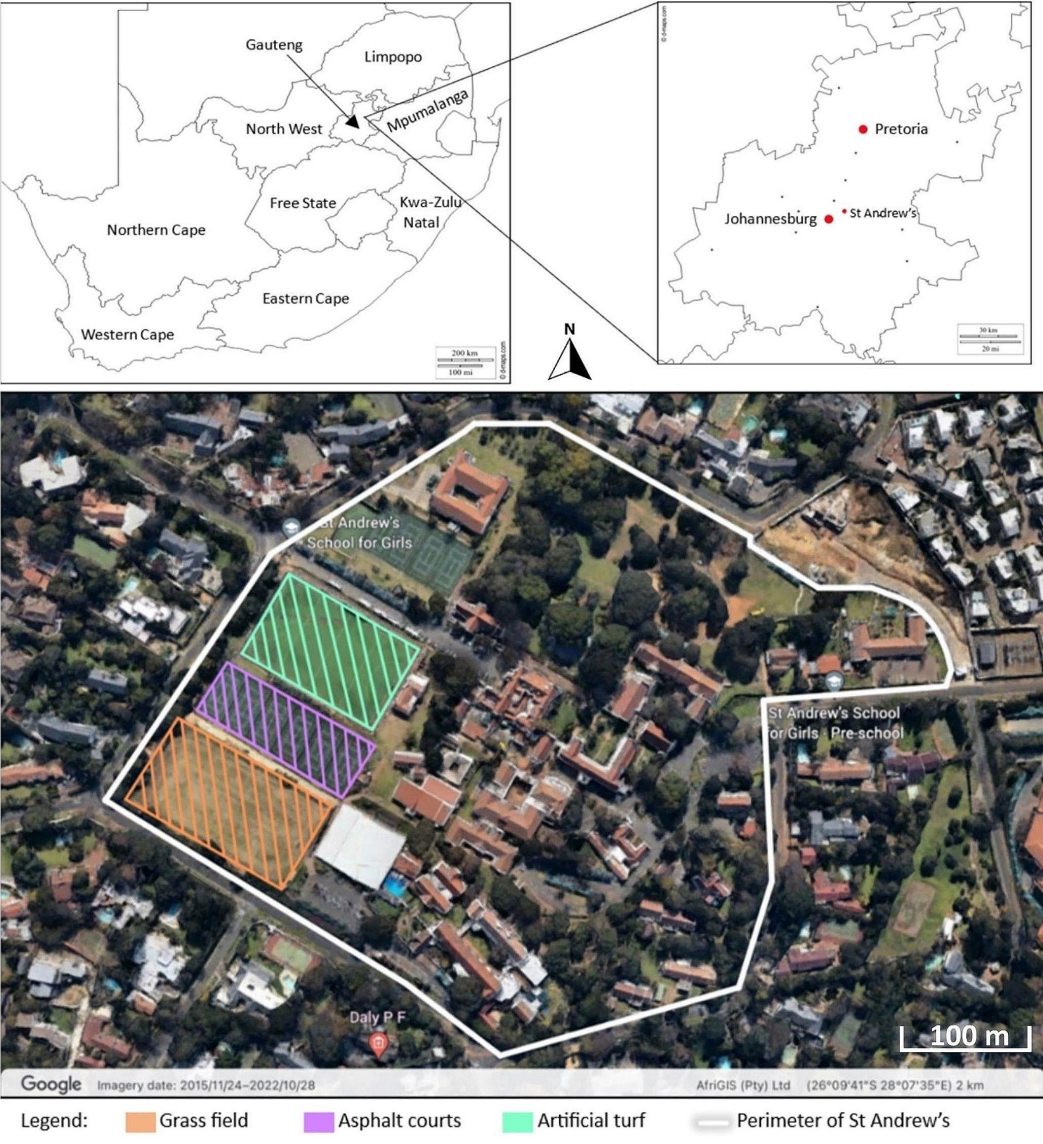
Figure showing map of South Africa, with the location of St Andrew’s School for Girls shown on a map of Gauteng, and an orthophoto of the school campus showing the three surfaces upon which data collection took place

This study is based in Johannesburg, Gauteng. More specifically, the site of this study is St Andrew’s School for Girls in Senderwood (Fig. 1). The school campus falls on the border of the City of Johannesburg and City of Ekurhuleni municipalities. Located at 26°09’S 28°07’E, the school is on the north-facing slope of Linksfield Ridge, with an altitude of approximately 1600 m.a.s.l (Fig. 1). The campus has a total area of 0.13km^2^, half of which is covered in vegetation; whilst the other half is covered by artificial surfaces, including asphalt, concrete, artificial turf. The school caters for learners between the ages of 3 months-18 years, but this study focusses on the Senior School, which encompasses Grades 8–12, with learner ages spanning 13–19 years. Several seasonal sports are offered to students, including tennis (September-March), athletics (September-October), cross-country (May-June), hockey, and netball (March-July). The school is ranked amongst the best girl’s schools in terms of sports performance (SASS 2023), and the competitive nature of sports results in an intensive training programme, which includes pre-season training. All sporting codes participate in interschool leagues and festivals. The sports facilities include a grass field, asphalt netball/tennis courts and an artificial turf, which is predominantly used for hockey. PE lessons take place from 07h30 to 14h30, and after-school sports activities take place from 14h30 to 20h00. These activities take place on all three above-mentioned surfaces. Since each of these three surfaces is likely to have different surface temperatures and specific microclimates, these surfaces form the specific monitoring sites for this study (Fig. 1).

Johannesburg is located in the north-eastern region of South Africa, with a local climate classification of subtropical highland, Köppen-Geiger climate classification Cwb (Peel et al. 2007). Johannesburg experiences four distinct seasons: summer, from September to the end of April; autumn in May; winter, in June and July; and spring in August (van der Walt and Fitchett 2020). Temperatures in Johannesburg peak during the midday hours between 11h00 and 14h00 (Kruger and Mbatha 2021). Historically, January, February, and December are the warmest months in Johannesburg, with a mean maximum of 28 °C (Kruger and Mbatha 2021). The highest air temperature (T_a_) recorded in Johannesburg is 35.6 °C at OR Tambo International Airport on the 7th of January 2016 (Kruger and Mbatha 2021). When defining a heat wave as “a period of three days or more with daily mean temperatures exceeding 5°C higher than the average of the hottest month”, Johannesburg can experience heat waves of up to 8 days long (Kruger and Mbatha 2021 p. 30). In terms of relative humidity, the annual average for Johannesburg is 60% (MetOffice.gov.uk; 2024). The summer months have a markedly higher relative humidity, with January being the highest at 73%. February, March, and December are all recorded to have relative humidity values above 70% (MetOffice.gov.uk). Winter months are recorded to have far lower levels of relative humidity, with the lowest relative humidity recorded in September, at 46% (MetOffice.gov.uk).

### Data collection

To assess the level of thermal comfort and heat stress, the following meteorological data were collected using a handheld Kestrel 5000 environmental monitor: T_a_(°C), relative humidity (RH; %), wind speed (v_a_; m.s^− 1^), wet-bulb temperature T_wb_;°C), whilst the surface temperature (T_surf_; °C) was measured with a Bosch UniversalTemp heat meter.

Data collection took place between the beginning of March and the end of July, which encompasses the pre-season and in-season training programmes for winter school sports. A total of 45 data collection sessions took place over the five months of the study, yielding 8100 individual meteorological readings, and 2700 individual heat stress index values. One to two sets of 12 data points were collected each day, during the times of a PE lesson (between the hours of 10h00 and 12h00), and the times of after-school sport sessions (between the hours of 14h30 and 17h30). Data collection took place in diverse weather conditions, to give realistic insight into the conditions under which sports take place at school. Meteorological measurements were taken on a grass field, a tennis/netball court, and an artificial turf to determine whether the surface type affects the potential for heat stress. Collection took place in the centre of the playing surfaces where possible, to mitigate the possible effects of wind breaks such as grandstands, and the influence of surrounding surfaces, and to best represent the conditions under which sports were being played. Data collection took place holding the Kestrel and Bosch radiant heat meter at approximately 1 m above the surface, which is a reasonable approximation of the average centre of mass of a human (S 7; Santee et al. 1994). Measurements were taken in situ over the course of an hour at 15-minute intervals. At the end of each interval, note was made of any potentially relevant observations, such as whether the surfaces were wet or dry, and the amount of cloud cover. This visual estimation of cloud cover was necessary for the use in some index calculators; and so clear conditions were classified as 0% cloud cover, cloudy conditions as 50% cloud cover, and overcast conditions as 100% cloud cover.

The meteorological data were screened for human error in reading and entering values. In two cases, outliers were identified and replaced with the average value of the other three readings taken during that session. These data were then grouped by surface type (grass, court, turf) per month (March, April, May, June, July). Sessions were classified according to the time of day at which data collection sessions ended: morning (end time from 08h00-11h00), midday (end time from 11h00-14h00), and afternoon (end time after 14h00). Meteorological data were averaged for each session, to provide an aggregate view of the weather in each hour. The maximum and minimum values were identified for each reading across each surface in each month.

### Data analysis

Four indices for thermal comfort were used: WBGT, Hx, HI, and UTCI. These indices produce values that determine the level of thermal comfort and express a level of risk for heat stress. Two classifications of Hx are used, shown as Hx1 and Hx2. Hx is typically applied in an occupational context where the level of physical activity and environmental acclimatisation is considered (OHCOW 2021). Hx1 is pertinent to those who are unfit and/or unacclimatised, whilst Hx2 is applied to those who are fit and/or acclimatised. For the UTCI, as mean radiative temperature (MRT) could not be measured at the time of the initial study, and as the playing surface was of primary concern as a source of radiative heat, the radiative heat measurements of the land surface were used in place of MRT. Following the completion of the study, MRT was later measured using a Kestrel 5400 device, and results compared to the UTCI calculated with radiative heat off the playing surface. The latter was found to over-estimate the effective temperature, but given the risk of the playing surface as a heat source, such over-estimation would be valuable in being more cautious in heat risk attribution. In addition to the four indices mentioned above, the T_wb_ was also recorded, which, although not a true heat stress index, gives a reasonable approximation of dangerous levels of heat stress, setting the threshold at which the interaction between T_a_ and RH results in uncompensable heat stress in humans (Vecellio et al. 2022; Vanos et al. 2023). Index values were calculated for each 15-minute reading and the output thermal stress risk quantified. The equations for each index and output classifications are presented in supplementary information 1–4.

The potential for heat stress was calculated by expressing the relative frequency of level of risk for each index as a percentage of the total number of readings. This allowed for the analysis of how different indices quantify heat stress differently, and indicates the sensitivity of each index to meteorological variables. Inter-index comparison was conducted, to establish the varying responses of indices to meteorological inputs. To circumvent the difficulty of comparing levels of risk for heat stress across indices that use different scales and evaluations of risk, each level of each index was categorised into ‘no risk’, ‘low risk’, ‘moderate risk’, ‘high risk’, ‘very high risk’, and ‘uncompensable heat stress’ (Table 1). This classification is based on the specific descriptions, recommendations and categorisations used by each index (S1-4). In doing so, the level of risk of heat stress can be compared across indices, to identify trends in conditions eliciting heat stress.

**Table 1:**
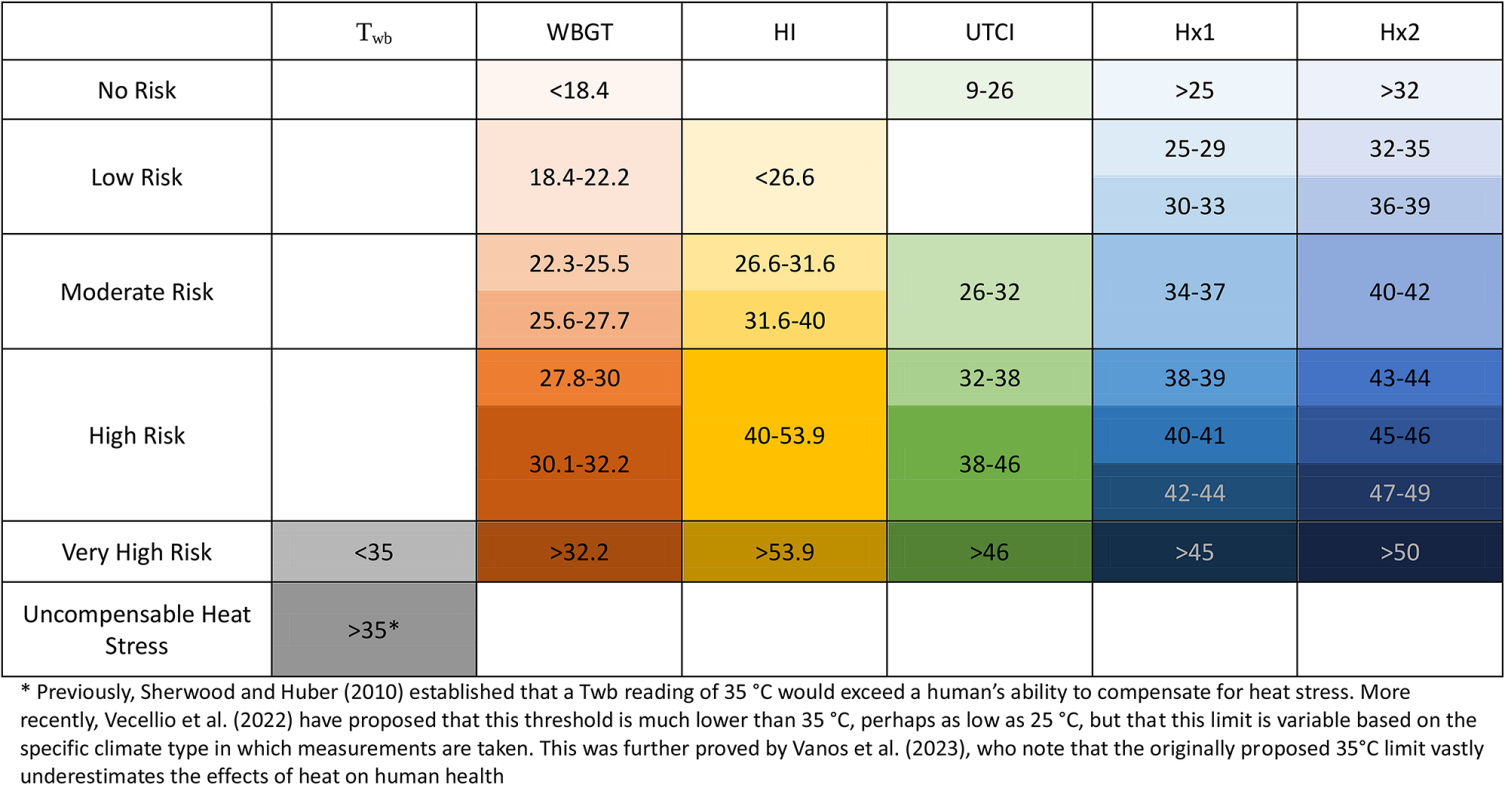
Classification of WBGT, HI, UTCI, Hx and T_wb_ index values into comparable levels

## Results

### Meteorological data

There is a considerable change in meteorological conditions between March and July, demonstrating the impact of seasonality. Average T_a_ (calculated per session) decreased from 27.9 °C in March to 15.6 °C in July. A similar decrease was observed in T_surf_ over the course of the study, from an average of 46.9 °C across surfaces in March to 29.2 °C in July. There is significant variation in T_surf_ across the three surfaces, with the highest T_surf_ values consistently recorded on the artificial turf, and the lowest on the grass field. The maximum T_surf_ values recorded on each surface are 44.5 °C on the grass field, 62.8 °C on the asphalt court, and 77.0 °C on the artificial turf, all recorded in March. RH levels decrease overall from March (average 39.9%) to July (average 32.4%), with higher RH generally recorded on the grass field, and the lowest values recorded on the asphalt court. There is no discernible pattern in the influence of seasonality on v_a_. Meteorological data show some response to the time of day. T_a_ generally increases between the morning and midday, and peaks in the afternoon sessions. Conversely, T_surf_ peaks around midday and early afternoon, and decreases steadily in the later afternoon. There is no clear relationship between the time of measurements and v_a_, but it generally decreases between midday and afternoon sessions. In all months except June, RH decreases as the day progresses, exhibiting a roughly indirectly proportional relationship to T_a_.

### Thermal index outputs

Overall, 540 calculations took place per index, resulting in a total of 2700 index values. Of these, 44.7% show no risk of heat stress, whilst 31.1% indicate a low risk of heat stress. A moderate level of risk is classified for 19.0% of index values. High risk and very high-risk incidents are not common, with only 5.6% of values indicating high risk conditions, and 0.3% of values suggesting very high risk of heat stress.

**Fig. 2:**
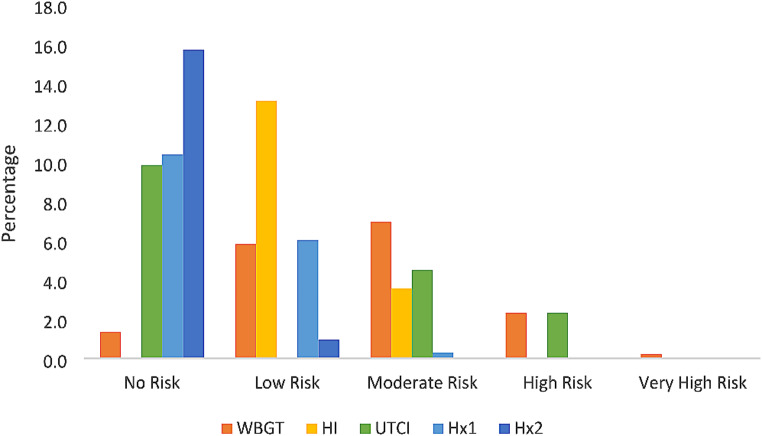
Relative frequency of index values by level of risk

In classifying the various descriptions of the levels of risk for each index, it becomes possible to observe that commonly used heat stress indices disagree on the level of risk presented by the same meteorological conditions (Simpson et al. 2023). Hx values indicate that the risk of heat stress throughout the study period is minimal, with the majority of readings indicating ‘no risk’ (Fig. 2). This is particularly the case when using Hx2, which is applied to those who are fit and acclimatised. ‘No risk’ readings also account for the majority of Hx1 and UTCI outputs. Conversely, WBGT returns a very small proportion of readings that present ‘no risk’ for heat stress. The absence of HI readings under ‘no risk’ conditions is attributable to the fact that the levels of classification of HI readings do not account for ‘no risk’; rather, any HI value less than 26.6 °C is considered ‘low risk’. HI readings most commonly produce ‘low risk’ outputs, with a smaller proportion of ‘low risk’ outputs resulting from WBGT and Hx1 readings. Here again, the lack of UTCI data under ‘low risk’ is a result of the index not accounting for that level of risk. The greatest number of ‘moderate risk’ outputs are attributable to WBGT, whilst HI and UTCI readings constitute the remainder, with a very small number of Hx1 readings producing ‘moderate risk’ values. Hereafter, only WBGT and UTCI account for the remaining levels of risk, with both indices producing an equal number of ‘high risk’ outputs, and WBGT producing the only ‘very high risk’ results (Fig. 2). With regards to the measurement of T_wb_ this study measured a maximum of 21.1 °C, recorded in March on the grass field, indicating no risk of uncompensable heat stress, which is defined by thresholds of 30–35 °C T_wb_.

**Fig. 3:**
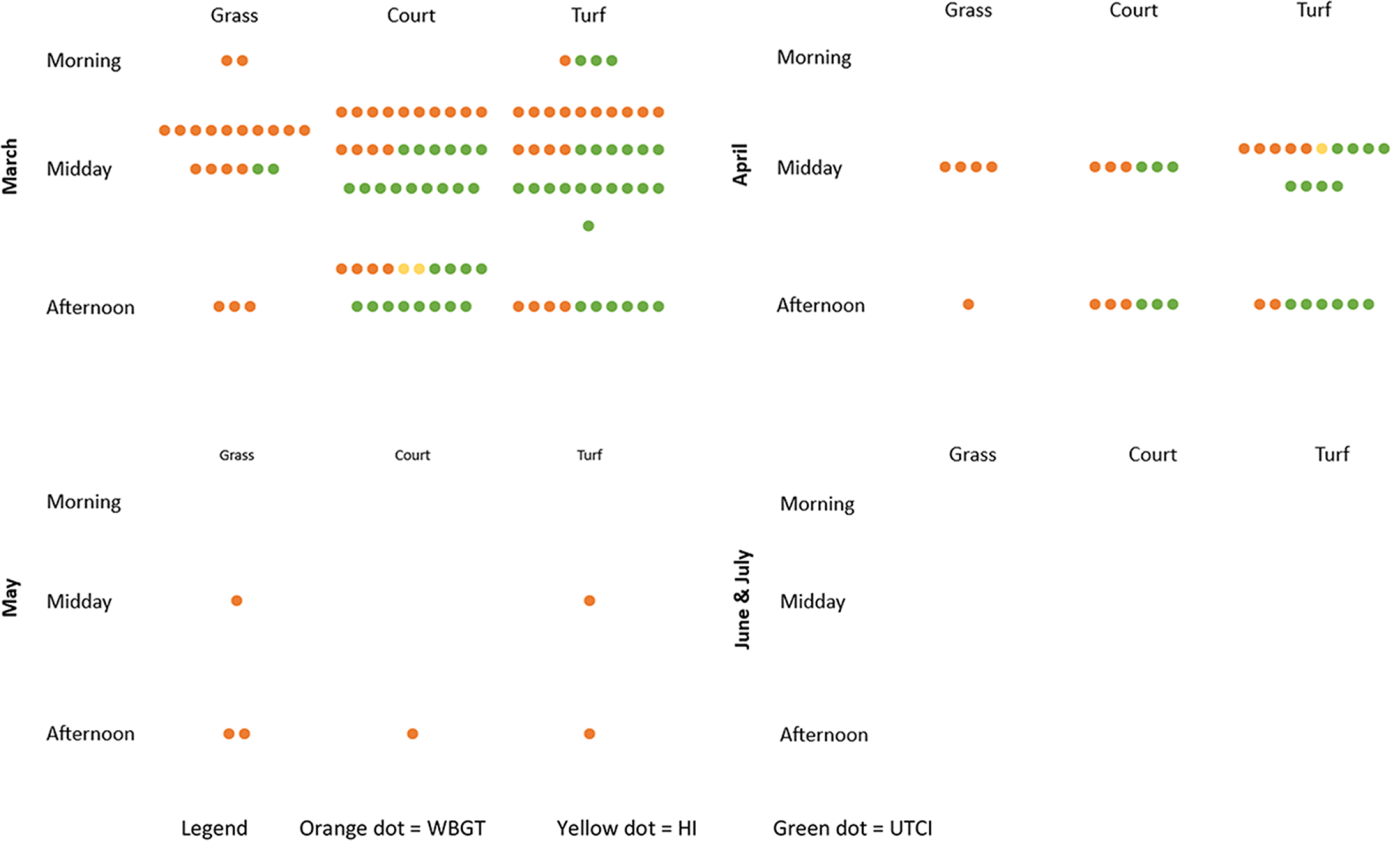
Occurrences of ‘high risk’ between March and July, across surface type and by time of day

In examining the specific incidence of ‘high risk’ and ‘very high risk’ results, it is possible to identify the spatial and temporal trends in the risk of heat stress. There is a clear seasonality in the occurrence of heat stress, with March exhibiting by far the highest risk (Fig. 3). A number of ‘high risk’ results are also recorded in April, and to a lesser extent in May. No incidences of ‘high risk’ are classified in June or July, but these months both recorded incidences of ‘moderate risk’. WBGT and UTCI are the two indices that most commonly produce results indicating higher levels of risk, which is likely a result of the indices’ inclusion of a greater number of meteorological variables, which produces a more accurate estimation of heat stress. WBGT results generally account for more ‘higher risk’ readings on the grass field, whilst UTCI account for more ‘higher risk’ readings on the artificial turf. It should be noted, however, that measuring T_surf_ in the instance of turf and court can lead to heightened output UTCI values, particularly under hot conditions, when compared to a true black bulb temperature record (S6).

## Discussion

### Variability of incidence

This study found notable variations in the meteorological variables, and consequently, the resultant heat stress index values, across time of day, seasonality, and surface type. In general, WBGT, UTCI, HI, and Hx values are highest during the midday period. Generally speaking, all four indices produce peak values around 13h00, and these values decrease fairly rapidly after 15h00, which corresponds to the findings of a study by Yeargin et al. (2023), and the reduction in incoming solar radiation through the afternoon. The decrease in the risk of heat stress is likely attributable to the decrease in T_a_ as the afternoon progresses, as well as the decrease in T_surf,_ both of which reduced the risk to the players (S5). This indicates that the risk of heat stress is greatest for students taking part in PE lessons during the middle of the school day, and sports activities that begin immediately after the school day ends (Fig. 3). Measurements taken before midday indicate that the risk of heat stress is generally far lower before 11h00, when T_a_ values are lowest. This underscores the importance of taking time of day into account when scheduling sports activities. Seasonal variations also influence the risk of heat stress; the risk of heat stress is far higher in the summer months than winter months. This is important to consider in light of the prevalence of pre-season training in many high-achieving schools which is intended to allow athletes to increase their fitness, skill, and mental preparedness before the official start of the season (Russell et al. 2022). However, a number of studies have shown that the risk of EHIs is greatest during the pre-season training period (Casa et al. 2015; Cooper et al. 2016; Kerr et al. 2019). Yeargin et al. (2016) observed the greatest occurrence of EHIs during the early autumn period, which is reflected by the results of this study. If November through April are considered to be summer months, it follows that a great deal of pre-season training takes place during the height of summer. Here there is consensus between indices, wherein WBGT, UTCI, and HI values are all highest in March, with a steady decrease throughout April (Fig. 3). However, even in May, there are instances of ‘high risk’ for heat stress according to WBGT values. This risk is compounded as generally athletes would be at their lowest fitness level prior to and at the beginning of a season.

The findings of this study align with findings of Kandelin et al. (1976) and Twomey et al. (2016): artificial turf tends to be far warmer than natural grass, and thus changes the heat risk profile between different playing environments. T_surf_ is an important contributing factor to heat stress, and in addition, also poses a risk for skin burns, given the high temperatures artificial turf reaches (Abraham 2019).Therefore, although the inclusion of T_surf_ results in higher UTCI values than black-bulb temperature, is arguably an important variable to include for this particular setting. The most substantial difference in maximum T_surf_ values across surfaces was recorded in March, at 32.5 °C variation between the grass field and artificial turf. Very high T_surf_ values were also recorded on the asphalt court, and this surface generally returned higher minimum values than the other two surfaces. Overall, the artificial turf exhibited the highest average T_surf_ values in most months, making the risk of heat stress higher on this surface. T_surf_ values recorded on the natural grass field are consistently the lowest of the three surfaces, indicating that the risk of heat stress is lower over this surface than the other two. In terms of index response to T_surf_, only the UTCI scores show a strong relationship between surface temperature and heat stress, likely due to the inclusion of T_surf_ in the equation. Of all UCTI values calculated, 62 indicated ‘strong heat stress’ and 13 indicated ‘very strong heat stress’. All of these were recorded on either the asphalt court or the artificial turf, with the majority occurring over the latter. The other indices used also indicate that the majority of higher levels of heat stress were recorded on either the asphalt court or the artificial turf, but the correlation is far less pronounced. A significant decrease in T_surf,_ and the associated UTCI values is noted in instances where the artificial turf is wet. In sessions where the turf was watered halfway through, a decrease of up to 17.3°C is observed, with a corresponding 6.5°C decrease in the UTCI value. However, it is difficult to ascertain the magnitude of the cooling effect caused by watering the turf, as there is a concurrent decrease in T_a_. This underscores the difficulty in quantifying the exact role that each meteorological variable plays in determining heat stress.

### Multi-index analysis

No index currently exists that is intended for use in high altitude sub-tropical conditions, such as those that exist in Johannesburg. And although WBGT, HI, Hx, and UTCI are four commonly used heat stress indices, none of these indices are designed specifically for use in sport. Furthermore, many of these indices have inherent assumptions relating to the gender, height, age and metabolism of the human subject (Grundstein and Vanos 2020). Despite this, the four indices used, as well as the T_wb_ reveal important dimensions of the risk of heat stress to those participating in physical exercise at school. Whilst all indices are inherently flawed in some way, using a multi-index approach allows for a reasonable estimation of the risk of heat stress (Grundstein and Vanos 2020). There is frequently consensus between the index outputs, where all four indices indicate some level of heat stress in a session. This is most common in March and April, where several indices values reach their peak in the same session. Whilst there are cases where the four indices, and the T_wb_ agree that there is a risk of heat stress, the exact level of risk is disputed in the index values. In the same session, for example, WBGT indicates a ‘very high risk’ of heat stress, whilst HI and Hx indicate that there is ‘low risk’, and UTCI indicates a ‘moderate risk’ of heat stress.

A further challenge in using a multi-index approach is the difficulties associated with comparing results. Even though some indices produce values in the same unit, e.g. °C, most indices function on their own individual scale (Simpson et al. 2023). This poses two difficulties; firstly, values may be confusing for users, particularly in the case of indices such as WBGT and T_wb_, where resultant values are relatively low. Data taken on the artificial turf frequently produced WBGT and T_wb_ values lower than the T_a_ (S5), which may lead users to believe that the risk of heat stress is low. These results are deceptive to those not familiar with the indices and their classifications (d’Ambrosio Alfano et al. 2014). Moreover, this overlooks the impact of direct skin exposure to very hot surface temperatures when learners are sitting on these surfaces or participating in sports barefoot. Secondly, the differing scales used by indices make it difficult to compare results directly. For example, an effective temperature of 30 °C represents serious heat stress by WBGT standards, where activity should be ceased for unfit athletes (Armstrong et al. 2007). However, based on Hx, a value of 30 °C represents a low risk of heat stress, wherein active workers are advised to increase water consumption (CCOHS 2023). A 30 °C value on the HI scale indicates that fatigue is possible (NOAA 2023), whilst UTCI categorises 30 °C as moderate heat stress (Błażejczyk et al. 2013). Calculating the risk of heat stress is complex and nuanced, compounded by the lack of a sports-specific heat stress index.

The absence of such an index should certainly not preclude heat stress monitoring at schools. This study has shown that a multi-index approach is a viable means of determining the risk of heat stress. However, this is a labour-intensive method, which may not be feasible for schools. A simplified approach to estimate heat stress by using easily acquired meteorological data is thus valuable. This study demonstrated that although different meteorological variables considerably influence the risk of heat stress, it is possible to identify the lowest T_a_ threshold at which heat stress is likely to occur. In comparing the incidence of the higher index values, it was found that when the T_a_ is 27.0 °C, 27.8 °C, and 28.2 °C on the grass, court, and turf respectively, all four indices indicate relatively high risk of heat stress (S5). This is a simplistic means of identifying the risk of heat stress as an easily employed monitoring strategy, that simply requires a thermometer to be mounted permanently next to each surface type, and checked prior to and during training to determine whether there is a risk of heat stress.

Casa et al. (2015) maintain that EHI is entirely preventable with proper monitoring and management strategies. Therefore, it is vital that schools consider the implications of heat stress for their students, and take measures to mitigate the risks of heat stress. Regardless of whether a school chooses to make use of an established index, or use T_a_ to estimate the risk of heat stress, it is also necessary for a school to enact other mitigation measures, to further reduce the risk of heat stress to students. Schools could introduce policies and guidelines related to heat safety. Here it is useful to look to the work done in the USA and Australia, where guidelines exist to inform teachers and coaches of the actions to be taken at each level of risk (Bergeron et al. 2011; Hyndman 2017; Yeargin et al. 2023). Increasing rest breaks in between periods of activity and encouraging students to increase their fluid intake are useful mitigation strategies at lower levels of risk (Hyndman 2017), as are the practices of pre- and percooling.

Considering that this study found that the risk of heat stress was markedly higher in March and April, one of the most vital considerations is the scheduling of pre-season training programmes. Pre-season training can start as early as November of the preceding year, and it is assumed that if there is high risk of heat stress in March, there is a considerable risk in November through February in the height of summer. It would be advisable for schools to schedule their pre-season sessions early in the morning or later in the afternoon when the risk of heat stress is lowest. Adams et al. (2021) also stress the importance of proper acclimatisation in minimising the risk of heat stress. Schools should therefore also include a carefully planned acclimatisation programme into their pre-season training, which allows for the measured exposure of athletes to heat during exercise. Schools should also consider the effect of surface type on heat stress (Kandelin et al. 1976), and adjust the location of their sports activities where possible. Consideration should be given to the design of sports kit, as clothing insulation can have an impact on the likelihood of heat stress (Hyndman 2017). Clothing that freely allows for evaporation of sweat is ideal, as it minimises the restriction of evaporative heat loss (Havenith 2002; Bergeron et al. 2011). Sports kit that is minimally insulative and simultaneously reflects incoming radiative heat would be best in the interests of keeping students cool during exercise (Havenith 2002).

## Conclusion

This study echoes the sentiments of many other researchers working in the field of exercise-induced heat stress in humans – there is substantial need for a heat stress index that can be applied to situations in which increased metabolic rates are accounted for. However, at the same time, there is also a need for a simpler index, which is easy to use and does not require specialised equipment. Herein lies another complexity in measuring heat stress, as these two needs are contradictory. Additionally, given the physiological differences between children, adolescents, and adults, it would be worth investigating whether separate heat stress indices are necessary for different age groups. And in light of the findings of this study, it seems imperative that models and indices that are designed to be used in a sport setting should account for the temperature of the surface upon which exercise is taking place. Heat stress is becoming an increasingly concerning threat to human health, particularly in light of projected climate change. As such, it is important to have the ability to monitor the risk of heat stress accurately and timeously during exercise in the interests of safeguarding athletes and students.

## Electronic supplementary material

Below is the link to the electronic supplementary material.

Supplementary Material 1

## Data Availability

All data are contained in the supplementary material.
