# Supplementary material for: Exploring the risk of heat stress in high school pre-season sports training, Johannesburg, South Africa
Source: Int J Biometeorol. 2024 Aug 14;69(11):2829–39. doi: 10.1007/s00484-024-02748-9 (PMC12540603; doi:10.1007/s00484-024-02748-9)

Exploring the risk of heat stress in high school pre-season sports training, Johannesburg, South Africa

International Journal of Biometeorology

Kayleigh Raines and Jennifer M. Fitchett*

School of Geography, Archaeology and Environmental Studies, University of the Witwatersrand, Private Bag X3, Wits 2050, South Africa

* Corresponding author: Jennifer.Fitchett@wits.ac.za

SUPPLEMENTARY INFORMATION

S1.a. Equation for WBGT

$$WBGT = 0.7Tnw + 0.2Tg + 0.1Ta*$$

Where: T_{nw} = natural wet bulb temperature (°C)

T_g = *black globe temperature* (°C)

T_a = *air temperature* (°C)

* For the purpose of this study, the above equation was not used, owing to the impracticality of a black globe thermometer. Instead, the online WBGT calculator available at https://wbgt.app was used to calculate WBGT values.

S1.b. WBGT risk categories for exercising individuals, in both continuous and intermittent activities (Adapted from Armstrong *et al*. (2007).

WBGT range °C	Continuous Activities	Intermittent Activities[1]	Intermittent Activities[2]
18.4-22.2	Risk of heat stress increases, monitor high risk individuals	Increase rest: work ratio, monitor fluid intake	Normal activity
22.3-25.5	Risk for all competitors is increased	Increase rest: work ratio, decrease total duration of activity	Monitor fluid intake
25.6-27.7	Risk for unfit. Non-acclimatised individuals is high	Decrease intensity and total duration of activity	Monitor fluid intake
27.8-30	Cancel activity/competition	Increase rest: work ratio to 1:1, limit intense exercise, monitor individuals carefully	Plan intense/prolonged activity with discretion, monitor individuals
30.1-32.2		Cancel or stop practice and competition	Limit intense exercise, monitor for signs of heat stress
>32.2		Cancel exercise	Cancel exercise
		[1] *Unfit/unacclimatised athletes*	[2] *Fit/acclimatised athletes*

S2.a. Equation for HI

$HI = -42.379 + 2.04901523Ta + 10.14333127RH - 0.22475541Ta.RH - 6.83783x10^{-3}Ta^2 - 5.481717x10^{-2}RH^2 + 1.22874x10^{-3}Ta^2 + 8.5282x10^{-4}Ta.RH^2 - 1.99x10^{-6}Ta^2RH^2*$

Where: T_a = air temperature (°F)

RH = relative humidity (%)

*The above equation is not able to accurately estimate a HI value for lower T_a and RH conditions and only produces accurate results where the HI is above 80°F. In such a case, it becomes necessary to use a simplified formula to calculate HI (NOAA, 2022). If this simplified equation returns a resultant value of greater than 80°F, the variables should only then be inputted into the above-given regression. The simplified equation is as follows:

$$HI = 0.5\ x\ \{Ta + 61.0 + [(Ta - 68.0)x1.2] + (RHx0.094)\}$$

Where: T_a = air temperature (°C)

RH = relative humidity (%)

S2.b. HI risk classification (NOAA, 2023).

HI range (°C)	General effect on people in high-risk groups
<26.6	Low risk
26.6-31.6	Fatigue possible with prolonged exposure or physical activity
31.6-40	Sunstroke, heat cramps, heat exhaustion possible with prolonged exposure or physical activity
40-53.9	Sunstroke, heat cramps, heat exhaustion likely with prolonged exposure or physical activity
>53.9	Heat/sunstroke highly likely with continues exposure

S3.a. Equation for Hx

$$Hx = Ta + \frac{5}{9}(e - 10)$$

Where: T_a = air temperature (°C)

$e = 6.112 \text{ x } 10^{(7.5Ta/237.7+Ta)} \text{ x } \frac{RH}{100}$

RH = relative humidity (%)

S3.b. Hx ranges for physical work with associated responses (CCOHS, 2023).

Hx 1 Range[1]	Recommended Response	Hx 2 Range[2]
25-29	Supply water to workers on an "as needed" basis	32-35
30-33	Heat Stress Alert notice<br>Encourage workers to drink extra water<br>Start recording hourly temperature & relative humidity	36-39
34-37	Heat Stress Warning notice<br>Notify workers that they need to drink extra water<br>Ensure workers are trained to recognise symptoms	40-42
38-39	Rest : work ratio = 15min/hour<br>Provide adequately cool water at a rate of 240ml per 20 minutes<br>Workers with symptoms should seek medical attention	43-44
40-41	Rest : work ratio = 30min/hour<br>In addition to provisions listed above	45-46
42-44	Rest : work ratio = 45min/hour<br>In addition to provisions listed above	47-49
>45	Only medically supervised work can continue	>50

[1] *Unacclimatised workers/moderate work*   [2] *Acclimatised workers/light work*

S4.a. Equation for UTCI

$$UTCI\ (Ta, MRT, va, RH) = Ta + Offset(Ta, MRT, va, RH)*$$

Where: T_a = air temperature (°C)

MRT = mean radiant temperature (°C)

v_a = wind speed ($m.s^{-1}$)

RH = relative humidity (%)

* The complexity of the UTCI equation necessitated the use of a pre-programmed online UTCI calculator, (available at https://citizenscienceproject.org.au/resources-for-citizens/thermal-comfort-tool/).

S4.b. UTCI equivalent temperature ranges categorised by the degree of thermal stress (Błażejczyk *et al.*, 2013)

UTCI (°C) range	Thermal stress category
> +46	Extreme heat stress
+38 to +46	Very strong heat stress
+32 to +38	Strong heat stress
+26 to +32	Moderate heat stress
+9 to +26	No thermal stress
0 to +9	Slight cold stress
-13 to 0	Moderate cold stress
-27 to -13	Strong cold stress
- 40 to -27	Very strong cold stress
< -40	Extreme cold stress

S5.a: Average, maximum and minimum T_a readings on grass, court and turf by month. Error bars represent the maximum and minimum values recorded per month per surface.

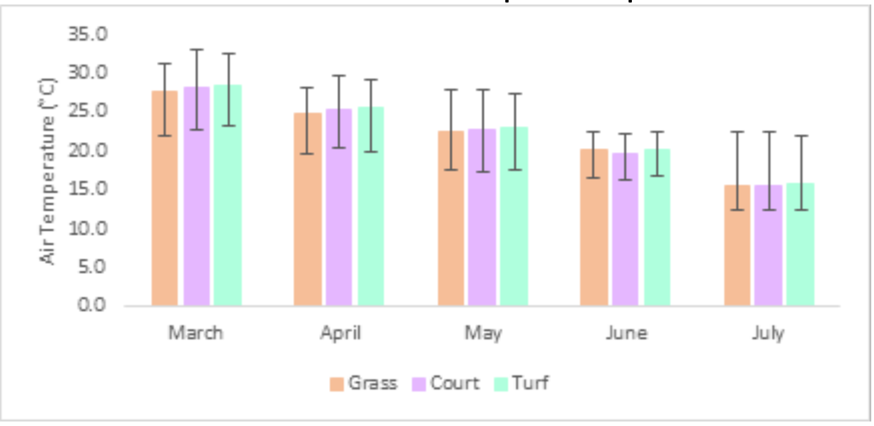

S5.b: Average, maximum and minimum T_{surf} readings on grass, court and turf by month. Error bars represent the maximum and minimum values recorded per month per surface.

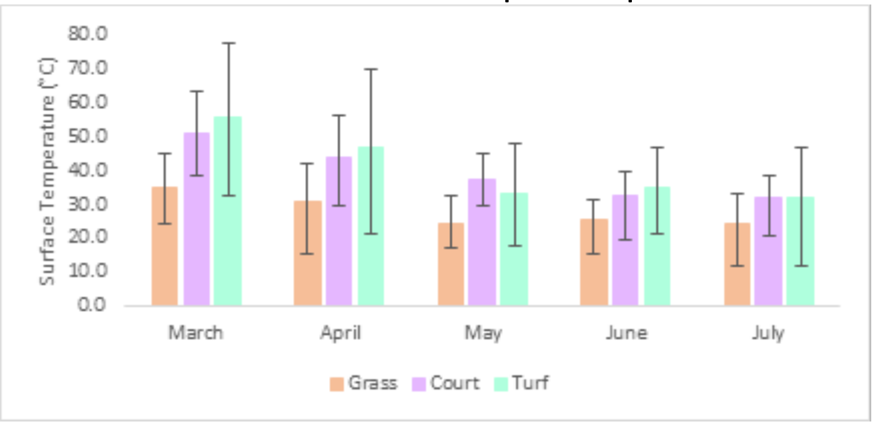

S5.c: Average, maximum and minimum RH readings on grass, court and turf by month. Error bars represent the maximum and minimum values recorded per month per surface.

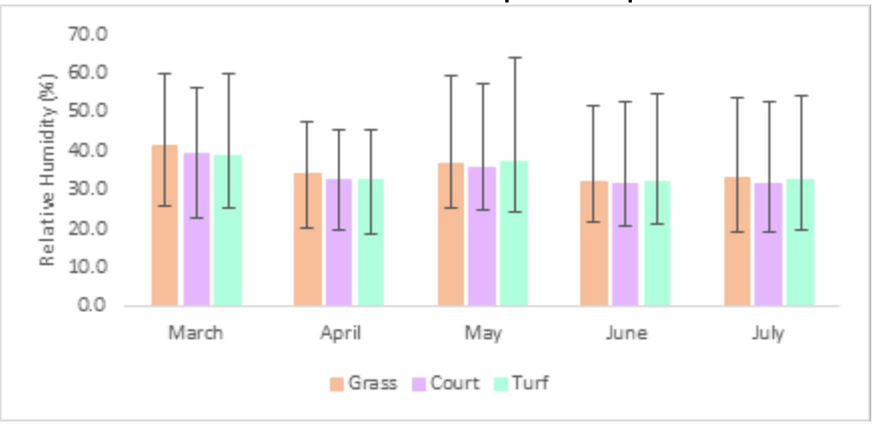

S5.d: Average, maximum and minimum v_a readings on grass, court and turf by month. Error bars represent the maximum and minimum values recorded per month per surface.

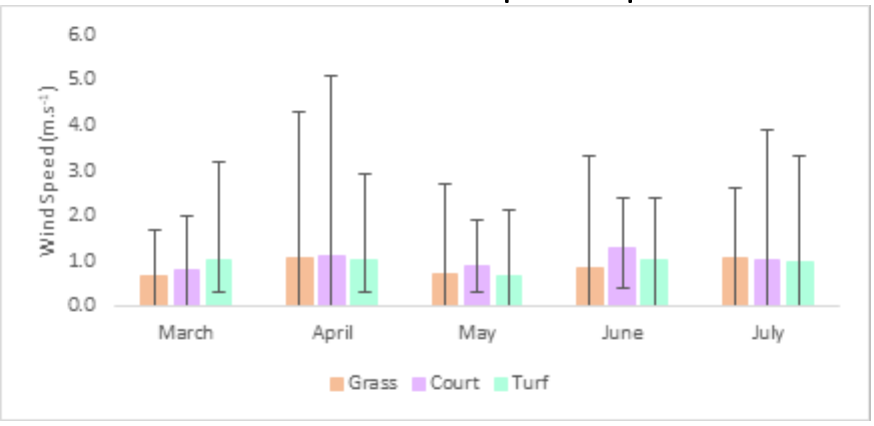

S5.e: Number of readings per month taking place under clear, cloudy and overcast conditions.

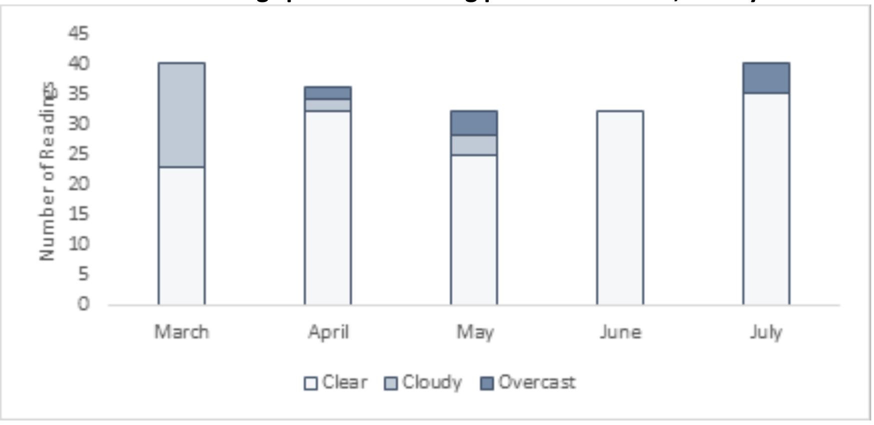

S5.f: Average, maximum and minimum T_{wb} readings on grass, court and turf by month. Error bars represent the maximum and minimum values recorded per month per surface.

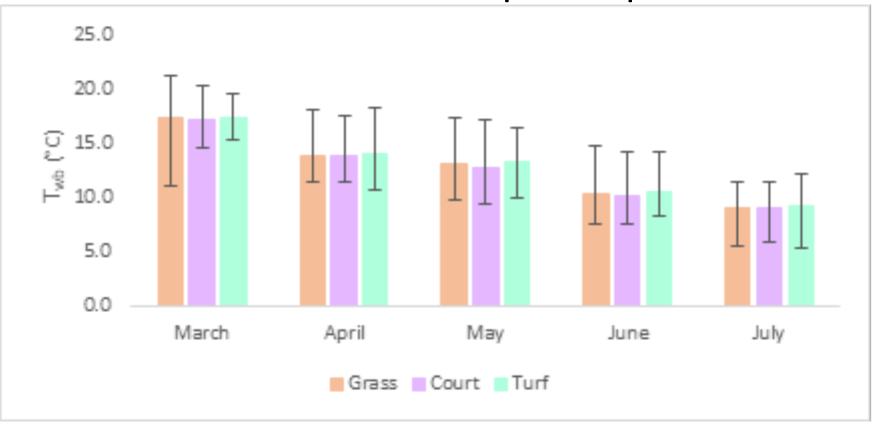

S5.g Meteorological data and output index data

		ID	Time	Grass										Court										Turf											
				Air Temperature °C	Surface Temperature °C	Humidity %	Wind Speed m/s	Wet Bulb	WBGT °C	Heat Index °C	UTCI °C	Humidex 1 °C	Humidex 2 °C	Air Temperature °C	Surface Temperature °C	Humidity %	Wind Speed m/s	Wet Bulb	WBGT °C	Heat Index °C	UTCI °C	Humidex 1 °C	Humidex 2 °C	Air Temperature °C	Surface Temperature °C	Humidity %	Wind Speed m/s	Wet Bulb	WBGT °C	Heat Index °C	UTCI °C	Humidex 1 °C	Humidex 2 °C	Notes	Sky Condition
March	Midday	1	3/3/23 11:44:01	24.4	30.3	56.0	0.9	17.9	29.0	25.8	26.1	28.3	28.3	24.8	43.2	56.0	2.0	17.0	24.8	26.3	28.8	29.0	29.0	24.0	54.0	54.5	2.4	18.4	23.2	25.3	30.4	27.5	27.5	Dry turf	Cloudy
		2	3/3/23 11:56:15	24.6	34.4	55.0	1.5	18.4	25.4	26.0	26.8	28.5	28.5	24.8	44.6	53.5	1.5	17.5	25.1	26.1	29.7	28.5	28.5	24.2	60.2	57.0	1.8	17.7	23.6	25.7	33.1	28.2	28.2	Dry turf	Cloudy
		3	3/3/23 12:08:30	24.1	28.7	58.9	1.7	18.0	25.1	25.6	24.8	28.3	28.3	25.7	46.7	54.3	0.6	17.9	28.4	26.5	31.4	30.1	30.1	23.3	44.6	59.7	2.9	16.7	23.7	24.8	26.8	27.2	27.2	Wet turf	Cloudy
		4	3/3/23 12:21:44	23.0	26.5	59.9	0.9	18.1	25.8	24.5	24.1	26.8	26.8	24.6	44.0	54.6	1.8	17.2	24.8	26.0	29.1	28.4	28.4	24.4	42.9	55.1	3.2	17.7	31.3	25.8	26.6	28.2	28.2	Wet turf	Cloudy
				**24.0**	**30.0**	**57.5**	**1.3**	**18.1**	**26.6**	**25.5**	**25.5**	**28.0**	**28.0**	**25.0**	**44.6**	**54.6**	**1.5**	**17.4**	**25.5**	**26.4**	**29.9**	**29.0**	**29.0**	**24.0**	**50.4**	**56.6**	**2.6**	**17.6**	**24.2**	**25.4**	**29.2**	**27.8**	**27.8**		
	Afternoon	5	3/3/23 14:37:48	26.5	35.1	49.0	1.0	18.0	26.7	26.9	28.6	30.3	30.3	25.5	47.9	49.4	0.6	17.0	29.3	26.5	31.3	28.9	28.9	29.3	59.2	45.3	0.3	19.6	23.3	29.5	37.0	34.0	34.0	Dry turf	Cloudy
		6	3/3/23 14:46:08	29.0	37.6	44.0	0.7	19.0	28.8	29.0	31.0	33.2	33.2	27.8	50.4	48.0	0.6	19.0	28.5	26.5	33.8	32.2	32.2	25.4	53.0	50.1	1.9	18.1	25.3	26.1	31.7	28.9	28.9	Wet turf	Cloudy
		7	3/3/23 14:58:08	25.0	35.0	55.0	0.9	17.3	26.1	26.4	27.8	29.1	29.1	23.8	41.2	54.0	1.8	17.1	23.2	25.1	27.6	27.1	27.1	24.8	41.6	51.5	2.4	17.9	25.4	26.0	27.6	28.2	28.2	Wet turf	Cloudy
		8	3/3/23 15:15:11	26.4	30.5	50.2	0.8	19.5	26.7	26.9	27.3	30.4	30.4	28.3	47.7	46.4	0.7	19.3	27.7	26.5	33.2	32.6	32.6	27.0	63.5	48.4	1.6	17.9	25.9	27.3	35.8	31.0	31.0	Wet turf	Cloudy
				**26.7**	**34.6**	**49.6**	**0.9**	**18.5**	**26.1**	**27.1**	**27.9**	**30.8**	**30.8**	**26.4**	**46.8**	**49.5**	**0.9**	**18.1**	**26.3**	**25.7**	**31.7**	**30.2**	**30.2**	**26.6**	**54.3**	**48.8**	**1.6**	**18.4**	**31.1**	**27.0**	**33.2**	**30.5**	**30.5**		
	Midday	9	3/9/23 11:43:09	30.6	40.5	36.0	0.0	19.0	33.7	29.9	27.6	33.8	33.8	30.0	61.0	31.0	1.0	17.7	27.8	28.8	37.0	31.7	31.7	28.9	60.5	33.3	1.8	17.0	25.2	28.0	35.6	30.7	30.7	Dry turf	Clear
		10	3/9/23 11:52:50	29.4	35.3	32.8	0.4	17.4	30.3	28.4	29.9	31.3	31.3	31.4	62.8	24.8	0.8	16.3	28.5	29.7	38.2	32.2	32.2	31.7	72.7	26.0	0.4	16.8	30.1	30.1	40.9	32.9	32.9	Dry turf	Clear
		11	3/9/23 12:05:15	29.0	32.9	26.8	1.0	15.7	26.6	27.7	28.6	29.4	29.4	31.0	52.7	29.5	0.6	17.2	29.9	28.1	35.6	32.8	32.8	29.2	68.1	26.4	1.8	15.6	27.3	27.8	37.5	29.6	29.6	Dry turf	Clear
		12	3/9/23 12:19:32	30.2	36.4	26.7	0.9	15.9	27.7	28.7	30.5	31.0	31.0	30.5	61.0	25.8	1.4	16.6	26.6	28.9	36.9	31.2	31.2	31.1	72.2	26.9	0.5	16.8	27.1	29.6	40.4	32.3	32.3	Dry turf	Clear
				**29.8**	**36.3**	**30.6**	**0.6**	**17.0**	**29.2**	**28.6**	**30.4**	**31.4**	**31.4**	**30.7**	**59.4**	**27.8**	**1.0**	**17.0**	**28.1**	**28.5**	**36.9**	**32.0**	**32.0**	**30.2**	**68.4**	**28.2**	**1.1**	**16.6**	**25.1**	**28.8**	**38.8**	**31.4**	**31.4**		
	Afternoon	13	3/9/23 15:56:11	29.4	32.0	32.6	0.9	17.7	25.8	28.4	29.0	31.3	31.3	33.1	51.5	30.0	0.4	19.9	30.1	32.1	37.0	36.0	36.0	32.4	35.0	28.9	1.2	18.5	24.2	31.1	32.1	34.6	34.6	Wet turf	Clear
		14	3/9/23 16:02:57	30.8	32.3	29.6	0.9	17.5	26.3	29.5	30.1	32.5	32.5	31.5	52.7	27.5	0.5	16.8	27.8	28.8	35.9	33.0	33.0	31.3	32.5	26.8	1.8	17.5	24.1	29.8	30.1	32.5	32.5	Wet turf	Clear
		15	3/9/23 16:08:50	30.7	31.0	30.5	0.4	17.8	28.0	29.5	29.7	32.6	32.6	32.7	50.5	35.5	0.4	19.5	30.2	32.5	36.8	36.9	36.9	29.7	39.9	28.5	1.7	16.8	25.2	28.4	30.8	30.7	30.7	Wet turf	Clear
		16	3/9/23 16:33:11	29.9	28.4	29.3	0.6	16.7	25.5	28.6	28.3	31.2	31.2	30.4	47.0	27.7	1.0	16.8	24.8	29.7	33.5	31.5	31.5	30.5	34.0	26.0	1.4	16.4	27.3	28.9	29.9	31.2	31.2	Wet turf	Clear
				**30.2**	**30.9**	**30.5**	**0.7**	**17.4**	**26.4**	**29.0**	**29.1**	**31.7**	**31.9**	**31.9**	**50.4**	**30.2**	**0.6**	**18.3**	**28.1**	**28.9**	**35.8**	**33.7**	**34.3**	**31.0**	**35.4**	**27.6**	**1.5**	**17.3**	**26.7**	**29.5**	**25.4**	**32.3**	**32.3**		
	Morning	18	3/16/23 9:49:00	22.0	33.4	55.0	0.5	15.5	25.2	23.1	25.1	24.5	24.5	22.8	40.7	51.0	0.5	15.9	25.5	23.8	27.6	25.1	25.1	23.2	40.3	52.1	0.3	15.3	26.4	24.3	27.8	25.9	25.9	Dry turf	Cloudy
		19	3/16/23 9:54:36	23.1	29.8	51.6	0.8	11.0	24.6	24.2	24.7	25.6	25.6	23.3	41.9	51.6	0.4	14.6	26.6	24.4	27.9	25.9	25.9	25.2	57.1	47.6	0.6	16.5	28.4	26.3	33.7	28.1	28.1	Dry turf	Cloudy
		20	3/16/23 10:09:55	24.9	34.7	50.6	0.3	16.5	29.2	26.1	27.4	28.2	28.2	23.0	38.2	46.8	0.9	15.7	24.1	23.8	26.8	24.7	24.7	23.5	55.3	48.2	0.5	16.0	27.3	24.4	32.2	25.7	25.7	Dry turf	Clear
		21	3/16/23 10:17:57	26.6	35.3	50.0	0.4	18.6	29.7	27.1	28.8	30.7	30.7	24.8	43.8	48.5	0.9	15.7	25.8	25.9	29.7	27.7	27.7	25.2	62.4	47.8	0.4	15.3	29.7	26.3	35.1	28.1	28.1	Dry turf	Clear
				**24.2**	**33.3**	**51.8**	**0.5**	**15.4**	**27.2**	**25.3**	**26.5**	**27.2**	**27.2**	**23.5**	**41.2**	**49.5**	**0.7**	**15.5**	**25.5**	**24.5**	**28.1**	**25.9**	**25.9**	**24.3**	**53.8**	**48.9**	**0.5**	**15.8**	**28.7**	**25.3**	**32.4**	**26.9**	**26.9**		
	Midday	22	3/21/23 12:31:06	27.0	38.9	48.0	0.5	18.9	29.9	27.3	30.0	30.9	30.9	28.0	57.3	42.3	0.4	17.6	30.7	26.5	35.4	31.3	31.3	28.5	72.5	40.6	0.6	19.0	31.0	28.2	39.4	31.7	31.7	Dry turf	Clear
		23	3/21/23 12:37:39	29.6	41.4	42.0	0.4	19.6	31.8	29.5	32.3	33.7	33.7	29.3	59.1	36.0	1.1	16.9	27.8	28.6	36.2	31.9	31.9	29.5	77.0	38.9	1.0	19.0	31.9	29.0	41.1	32.8	32.8	Dry turf	Clear
		24	3/21/23 13:00:27	30.6	41.2	42.0	0.6	21.1	33.9	30.7	33.1	35.3	35.3	31.4	59.3	36.0	0.6	20.2	31.2	30.9	38.1	35.0	35.0	31.5	74.6	34.1	0.6	19.4	30.0	30.7	41.9	34.7	34.7	Dry turf	Clear
		25	3/21/23 13:20:09	29.8	34.2	41.3	0.0	19.6	33.8	29.6	30.6	33.8	33.8	31.0	58.8	38.7	0.4	19.1	32.3	30.7	37.8	35.1	35.1	30.7	75.5	36.6	0.4	19.5	26.4	30.1	41.6	34.1	34.1	Dry turf	Clear
				**29.3**	**38.9**	**43.3**	**0.4**	**19.8**	**32.1**	**29.2**	**31.5**	**33.5**	**33.5**	**29.9**	**58.6**	**38.3**	**0.6**	**18.5**	**30.2**	**28.1**	**36.9**	**33.3**	**33.3**	**30.1**	**74.9**	**37.6**	**0.7**	**19.2**	**24.2**	**29.5**	**41.0**	**33.4**	**33.4**		
	Afternoon	26	3/21/23 16:29:45	28.0	30.4	42.5	0.4	18.5	26.6	27.8	28.1	31.4	31.4	29.1	46.3	36.9	0.4	17.9	26.8	28.4	32.9	31.8	31.8	30.7	49.2	32.8	0.7	17.1	25.1	29.7	34.6	33.2	33.2	Dry turf	Cloudy
		27	3/21/23 16:39:40	27.7	28.6	39.5	0.8	17.6	24.6	27.4	27.1	30.3	30.3	28.9	44.5	40.5	0.5	18.1	26.3	28.5	32.5	32.3	32.3	28.8	42.3	37.5	1.5	18.0	25.9	28.2	31.3	31.5	31.5	Dry turf	Cloudy
		28	3/21/23 16:56:24	26.9	24.1	41.8	0.4	17.3	24.5	26.9	25.4	29.6	29.6	27.6	41.5	39.1	0.4	17.4	24.7	26.8	30.6	30.0	30.0	28.1	37.1	40.9	0.5	17.4	25.4	27.8	29.9	31.2	31.2	Dry turf	Cloudy
		29	3/21/23 17:00:20	27.3	24.3	40.9	0.8	17.5	23.5	27.1	25.7	30.0	30.0	27.6	40.6	39.6	0.5	17.7	24.3	28.8	30.4	30.2	30.2	29.2	36.1	39.6	0.4	17.9	30.6	28.8	30.4	32.5	32.5	Dry turf	Cloudy
				**27.5**	**26.9**	**41.2**	**0.6**	**17.7**	**24.8**	**27.3**	**26.6**	**30.3**	**30.3**	**28.3**	**43.2**	**39.0**	**0.5**	**17.8**	**25.5**	**29.7**	**31.6**	**31.1**	**31.1**	**29.2**	**41.2**	**37.7**	**0.8**	**17.6**	**29.2**	**28.6**	**31.7**	**32.1**	**32.1**		
	Midday	30	3/22/23 12:16:09	29.8	40.2	33.6	0.3	17.5	31.8	28.8	31.6	32.1	32.1	29.8	53.7	33.9	0.7	17.1	29.5	29.7	35.2	32.1	32.1	30.4	72.3	29.7	0.5	17.3	29.7	29.1	40.1	32.0	32.0	Dry turf	Clear
		31	3/22/23 12:29:59	27.5	40.8	31.0	1.3	16.0	25.4	26.8	27.2	28.3	28.3	29.3	57.4	33.0	0.5	16.5	29.8	28.9	35.8	31.2	31.2	29.5	70.8	26.7	0.5	15.8	29.5	28.1	38.9	30.0	30.0	Dry turf	Clear
		32	3/22/23 12:44:40	29.0	41.8	35.1	0.4	17.1	30.6	28.2	31.5	31.2	31.2	28.0	59.0	28.3	1.6	14.6	24.9	29.3	34.5	28.4	28.4	30.1	71.0	31.2	0.6	17.3	29.4	28.9	39.7	31.9	31.9	Dry turf	Clear
		33	3/22/23 12:56:38	29.7	38.0	31.2	0.5	16.9	29.9	28.6	30.8	31.4	31.4	28.7	59.1	30.8	0.8	14.9	27.6	27.7	35.6	29.9	29.9	30.9	69.6	26.3	0.6	16.6	27.7	29.3	39.6	31.9	31.9	Dry turf	Clear
				**28.7**	**40.2**	**34.4**	**0.6**	**17.0**	**28.8**	**27.9**	**30.8**	**30.6**	**30.6**	**28.8**	**54.5**	**33.0**	**0.8**	**16.2**	**28.0**	**30.0**	**34.6**	**30.5**	**30.5**	**30.0**	**65.0**	**30.3**	**0.6**	**16.9**	**27.8**	**28.8**	**38.0**	**31.6**	**31.6**		
	Afternoon	34	3/22/23 14:48:01	28.4	39.6	25.9	0.5	15.2	27.0	27.2	29.9	28.4	28.4	28.6	57.6	22.9	0.9	14.8	25.5	27.2	34.7	28.0	28.0	30.2	37.4	25.3	0.6	16.2	28.3	28.6	30.7	30.7	30.7	Wet turf	Cloudy
		35	3/22/23 15:00:20	31.3	36.1	25.7	0.3	17.0	30.4	29.7	31.3	32.3	32.3	29.6	59.0	22.5	1.0	15.0	25.6	29.0	35.7	29.2	29.2	29.9	35.8	27.1	0.6	16.4	29.7	28.5	30.1	30.7	30.7	Wet turf	Clear
		36	3/22/23 15:13:17	29.3	36.0	32.9	0.6	16.1	27.7	28.3	30.0	31.2	31.2	29.7	51.7	23.2	0.8	15.2	25.8	28.1	34.0	29.5	29.5	30.5	43.7	26.6	0.5	16.9	28.1	29.0	32.7	31.4	31.4	Wet turf	Cloudy
		37	3/22/23 15:19:45	27.9	27.1	28.0	0.9	15.0	24.8	26.9	26.2	28.2	28.2	29.5	43.8	30.4	0.0	16.7	30.2	28.3	32.1	30.9	30.9	30.9	43.1	37.0	0.6	18.0	27.8	30.4	33.5	34.5	34.5	Wet turf	Cloudy
				**29.2**	**34.7**	**28.1**	**0.6**	**15.8**	**27.1**	**28.0**	**29.3**	**30.0**	**30.0**	**29.4**	**53.0**	**24.8**	**0.7**	**15.4**	**26.3**	**27.9**	**34.2**	**29.4**	**29.4**	**30.4**	**40.0**	**29.0**	**0.6**	**16.9**	**29.7**	**29.0**	**31.8**	**31.8**	**31.8**		
	Midday	38	3/29/23 12:09:34	25.1	44.5	48.2	0.4	17.4	29.2	26.2	30.1	28.1	28.1	25.0	52.4	50.2	0.3	18.0	30.2	26.2	31.4	28.3	28.3	26.1	64.5	45.1	0.7	18.5	29.9	26.5	36.0	29.0	29.0	Dry turf	Clear
		39	3/29/23 12:27:14	25.0	36.6	54.2	0.7	17.4	27.8	26.4	28.2	29.0	29.0	25.8	53.1	48.4	1.0	16.8	30.8	26.5	32.9	29.2	29.2	25.9	65.0	47.0	0.4	16.7	27.9	26.4	36.2	29.1	29.1	Dry turf	Clear
		40	3/29/23 12:33:15	26.5	37.6	40.0	0.6	17.4	28.2	26.5	28.7	28.6	28.6	27.0	53.0	46.0	0.6	18.0	29.2	25.7	33.8	30.5	30.5	27.2	66.0	46.0	0.5	18.0	28.7	27.4	37.2	30.8	30.8	Dry turf	Clear
		41	3/29/23 12:49:21	26.9	38.6	48.2	0.3	16.6	31.4	27.2	29.8	30.8	30.8	26.0	54.3	51.0	0.5	18.9	29.4	25.9	33.7	29.9	29.9	26.3	64.7	45.5	0.7	17.4	28.6	26.6	36.2	29.4	29.4	Dry turf	Clear
				**25.9**	**39.3**	**47.7**	**0.5**	**17.2**	**29.1**	**26.4**	**29.2**	**29.1**	**29.1**	**26.0**	**53.2**	**48.9**	**0.6**	**17.9**	**28.7**	**26.2**	**33.3**	**29.5**	**29.5**	**26.4**	**65.1**	**45.9**	**0.6**	**17.7**	**28.8**	**26.7**	**36.4**	**29.6**	**29.6**		
			**Average**	**27.6**	**34.5**	**41.3**	**0.7**	**17.4**	**27.9**	**27.5**	**28.6**	**30.3**	**30.3**	**28.0**	**50.7**	**39.4**	**0.8**	**17.2**	**27.5**	**27.7**	**33.4**	**30.5**	**30.3**	**28.2**	**55.3**	**38.9**	**1.0**	**17.4**	**27.2**	**27.9**	**34.6**	**30.7**	**30.3**		

			**Maximum**	**31.3**	**44.5**	**59.9**	**1.7**	**21.1**	**33.9**	**30.7**	**33.1**	**35.3**	**35.3**	**33.1**	**62.8**	**56.0**	**2.0**	**20.2**	**32.3**	**32.5**	**38.2**	**36.9**	**36.9**	**32.4**	**77.0**	**59.7**	**3.2**	**19.6**	**31.9**	**31.1**	**41.9**	**34.7**	**34.7**		
			**Minimum**	**22.0**	**24.1**	**25.7**	**0.0**	**11.0**	**23.5**	**23.1**	**24.1**	**24.5**	**24.5**	**22.8**	**38.2**	**22.5**	**0.0**	**14.6**	**23.2**	**23.8**	**26.8**	**24.7**	**24.7**	**23.2**	**32.5**	**25.3**	**0.3**	**15.3**	**23.2**	**24.3**	**25.4**	**25.7**	**25.7**		
**April**	Midday	42	4/5/23 12:04:02	22.5	39.7	45.3	1.2	15.1	23.5	23.2	26.6	23.8	23.8	24.4	52.7	43.7	0.6	15.8	26.3	24.5	31.8	26.3	26.3	25.7	66.0	44.8	0.7	15.8	27.9	26.5	36.1	28.4	28.3	Dry turf	Clear
		43	4/5/23 12:19:03	27.9	38.7	43.0	0.0	18.1	33.0	27.8	30.3	31.3	31.3	27.2	52.1	39.4	0.4	16.3	32.7	31.2	33.3	29.5	29.5	26.7	69.0	40.5	0.6	16.7	30.5	29.2	37.3	29.0	29.0	Dry tur	Clear
		44	4/5/23 12:30:43	26.4	37.7	46.1	0.7	17.6	28.2	26.7	27.3	29.6	29.6	28.4	53.5	40.0	0.7	17.4	29.8	29.3	34.5	31.4	31.4	28.2	69.5	41.3	0.4	17.9	33.3	31.7	38.5	31.6	31.6	Dry turf	Clear
		45	4/5/23 12:44:25	25.5	30.7	45.4	0.3	16.7	30.1	26.5	26.4	28.2	28.2	23.7	52.0	41.3	0.4	14.2	30.6	27.9	31.2	24.9	24.9	25.9	67.4	41.3	0.3	17.1	29.1	25.6	36.5	28.1	28.1	Dry turf	Cloudy
				**25.6**	**36.7**	**45.0**	**0.6**	**16.9**	**28.3**	**26.5**	**28.1**	**28.2**	**28.2**	**25.9**	**52.6**	**41.1**	**0.5**	**15.9**	**29.8**	**27.9**	**32.7**	**28.0**	**28.0**	**26.6**	**68.0**	**42.0**	**0.5**	**16.9**	**30.1**	**27.8**	**37.1**	**29.3**	**29.3**		
	Afternoon	46	4/5/23 14:29:18	27.1	42.0	41.7	0.0	17.8	31.4	27.0	30.5	29.8	29.8	26.0	55.8	36.7	0.8	15.1	28.7	29.2	33.2	27.3	27.3	27.7	67.0	34.2	0.5	15.9	27.9	26.8	37.1	29.3	29.3	Dry turf	Clear
		47	4/5/23 14:38:47	25.4	27.6	41.2	0.4	16.4	22.1	26.1	25.1	27.3	27.3	25.9	46.7	38.8	0.5	15.0	23.1	27.0	30.7	27.5	27.5	25.5	43.1	39.7	0.3	15.8	23.9	27.3	29.5	27.1	27.1	Dry turf	Overcast
		48	4/5/23 15:04:24	23.3	23.7	47.6	0.5	15.5	20.5	24.2	22.8	25.3	25.3	23.9	38.1	42.6	0.5	15.0	21.6	26.1	27.2	25.4	25.4	23.5	48.3	41.4	0.7	13.5	21.1	26.1	29.7	24.5	24.5	Dry turf	Overcast
		49	4/5/23 15:18:16	25.0	33.4	44.4	0.5	17.0	26.3	25.9	26.7	27.2	27.2	26.1	42.0	35.2	0.5	14.2	26.9	26.8	29.4	27.1	27.1	27.6	50.8	37.8	0.5	17.4	27.2	26.9	33.1	29.8	29.8	Dry turf	Cloudy
				**25.2**	**31.7**	**43.7**	**0.4**	**16.7**	**22.2**	**26.1**	**26.3**	**27.4**	**27.4**	**25.5**	**45.7**	**38.3**	**0.6**	**14.8**	**22.8**	**27.1**	**30.2**	**26.8**	**26.8**	**26.1**	**52.3**	**38.3**	**0.5**	**15.7**	**27.5**	**26.7**	**31.0**	**27.6**	**27.6**		
	Afternoon	50	4/7/23 14:40:27	25.5	35.5	34.4	1.0	14.8	24.5	25.9	27.1	26.2	26.2	27.7	46.3	31.6	0.4	15.0	27.3	26.5	31.5	28.7	28.7	28.4	53.9	28.9	1.0	15.5	26.2	27.5	33.9	29.1	29.1	Dry turf	Clear
		51	4/7/23 14:55:27	25.9	33.6	30.1	0.6	13.4	25.6	26.1	26.7	25.9	25.9	29.5	45.0	30.0	0.6	16.7	25.6	26.1	32.4	30.8	30.8	27.9	49.0	37.6	0.7	18.2	29.6	30.3	32.7	30.3	30.3	Dry turf	Clear
		52	4/7/23 15:10:28	27.3	33.8	28.5	1.4	14.6	23.9	26.6	27.4	27.5	27.5	29.3	45.0	26.1	0.0	15.1	29.1	26.6	32.1	29.6	29.6	29.0	49.5	26.9	1.4	15.5	25.4	28.2	32.9	29.5	29.5	Dry turf	Clear
		53	4/7/23 15:26:03	27.1	33.0	27.3	2.1	13.8	22.6	26.4	26.3	27.0	27.0	28.8	45.7	24.4	0.5	14.7	25.6	26.2	31.8	28.6	28.6	28.7	52.1	23.8	0.6	14.2	26.1	27.3	33.5	28.4	28.4	Dry turf	Clear
				**26.5**	**34.0**	**30.1**	**1.3**	**14.2**	**23.7**	**26.1**	**27.0**	**26.7**	**26.7**	**28.8**	**45.5**	**28.0**	**0.4**	**15.4**	**27.2**	**26.1**	**31.9**	**29.4**	**29.4**	**28.5**	**51.1**	**29.3**	**0.9**	**15.9**	**26.6**	**28.2**	**33.3**	**29.4**	**29.4**		
	Afternoon	54	4/7/23 16:32:30	25.4	26.1	29.0	0.5	13.0	23.1	25.5	23.1	25.1	25.1	26.4	39.5	28.0	0.7	14.1	21.9	25.1	28.6	26.2	26.2	27.0	38.0	26.6	1.1	14.3	21.7	26.3	28.5	26.7	26.7	Dry turf	Clear
		55	4/7/23 16:46:30	26.9	24.0	28.4	0.0	14.5	25.4	26.3	24.6	26.9	26.9	26.2	37.6	28.2	0.8	14.4	22.6	26.3	27.9	26.0	26.0	27.6	33.8	27.5	0.5	14.3	22.7	26.1	27.8	27.9	27.9	Dry turf	Clear
		56	4/7/23 17:02:19	26.6	23.1	30.5	0.3	14.6	23.8	26.2	24.2	26.9	26.9	26.2	34.2	30.3	0.7	14.6	22.5	26.4	27.1	26.4	26.4	25.9	29.1	29.4	1.9	14.3	20.6	26.6	24.6	25.9	25.9	Dry turf	Clear
		57	4/7/23 17:15:28	25.3	20.4	32.3	0.8	14.6	20.8	25.6	22.5	25.5	25.5	25.5	31.7	32.6	0.5	15.2	21.7	25.8	25.9	25.8	25.8	25.3	26.3	33.0	0.5	14.3	22.0	26.2	24.3	25.8	25.8	Dry turf	Clear
				**26.1**	**23.4**	**30.1**	**0.4**	**14.2**	**23.4**	**26.3**	**23.9**	**26.1**	**26.1**	**26.1**	**35.8**	**29.8**	**0.7**	**14.6**	**22.1**	**26.3**	**27.4**	**26.1**	**26.1**	**26.5**	**31.8**	**29.1**	**1.0**	**14.3**	**21.5**	**26.3**	**26.5**	**26.6**	**26.6**		
	Midday	58	4/12/23 11:51:56	21.7	35.0	38.6	2.1	12.3	20.8	21.9	23.2	21.7	21.7	20.6	45.9	40.1	5.1	12.7	19.3	22.0	20.7	20.4	20.4	21.4	53.0	39.9	2.7	12.5	19.4	20.6	27.2	21.5	21.5	Dry turf	Clear
		59	4/12/23 12:08:37	22.5	35.1	35.1	2.5	12.6	20.7	22.6	23.0	22.3	22.3	22.0	46.3	36.7	2.0	11.9	21.2	22.4	26.8	21.8	21.8	23.0	59.0	37.4	2.7	12.5	20.3	22.0	29.9	23.3	23.3	Dry turf	Clear
		60	4/12/23 12:24:35	22.2	33.6	36.5	1.9	12.8	21.2	22.4	23.4	22.1	22.1	21.1	45.7	36.6	2.6	11.5	20.4	22.2	25.0	20.6	20.6	20.6	55.1	37.0	2.9	12.4	19.1	20.7	26.8	20.0	20.0	Dry turf	Clear
		61	4/12/23 12:43:46	22.5	34.2	42.0	1.7	13.9	22.3	23.0	24.3	23.3	23.3	22.2	46.2	40.4	3.9	12.9	21.1	23.8	23.9	22.6	22.6	22.2	52.1	40.8	1.5	13.1	22.6	23.1	29.4	22.8	22.8	Dry turf	Clear
				**22.2**	**34.5**	**38.1**	**2.1**	**12.9**	**21.2**	**22.5**	**23.4**	**22.3**	**22.3**	**21.5**	**46.0**	**38.5**	**3.4**	**12.3**	**20.3**	**22.6**	**24.1**	**21.4**	**21.4**	**21.8**	**54.8**	**38.8**	**2.5**	**12.6**	**20.3**	**21.6**	**28.3**	**21.9**	**21.9**		
	Afternoon	62	4/12/23 16:10:46	22.5	23.4	39.7	0.6	13.1	22.5	22.9	21.7	22.9	22.9	23.3	36.1	38.7	0.4	14.1	23.7	23.3	26.0	23.9	23.9	23.3	33.7	38.3	0.6	12.9	23.4	24.3	25.3	23.9	23.9	Dry turf	Clear
		63	4/12/23 16:25:05	22.4	19.0	41.0	0.7	13.2	21.6	22.8	20.3	23.0	23.0	22.0	33.1	40.1	0.5	12.8	22.8	23.5	24.4	22.3	22.3	23.2	31.5	40.4	0.5	14.8	22.3	22.7	24.7	24.0	24.0	Dry turf	Clear
		64	4/12/23 16:41:10	19.5	17.7	40.4	4.3	11.4	16.1	19.6	12.5	19.0	19.0	20.9	31.9	42.2	2.7	12.8	16.4	19.2	20.8	21.1	21.1	21.4	26.2	41.1	1.5	12.4	18.8	21.4	21.3	21.5	21.5	Dry turf	Clear
		65	4/12/23 16:55:41	19.6	15.4	45.9	1.8	12.0	17.4	20.0	16.4	19.9	19.9	20.3	29.4	45.1	1.0	12.4	18.4	20.2	24.8	20.7	20.7	19.9	21.4	45.5	1.2	12.5	18.8	21.2	19.3	20.1	20.1	Dry turf	Clear
				**21.0**	**18.9**	**41.8**	**1.9**	**12.4**	**18.7**	**21.3**	**18.2**	**21.2**	**21.2**	**21.6**	**32.6**	**41.5**	**1.2**	**13.0**	**19.6**	**21.5**	**23.8**	**22.0**	**22.0**	**22.0**	**28.2**	**41.3**	**1.0**	**13.2**	**21.1**	**22.4**	**22.9**	**22.2**	**22.2**		
	Midday	66	4/14/23 12:22:03	24.7	34.0	28.9	0.8	13.2	24.6	24.7	25.9	24.1	24.1	28.8	49.6	21.9	0.4	13.2	25.8	23.7	32.7	28.1	28.1	27.0	60.2	23.9	0.3	12.8	29.6	26.9	34.5	26.2	26.2	Dry turf	Clear
		67	4/14/23 12:30:28	24.8	34.5	23.3	1.5	11.8	22.2	24.6	25.5	23.3	23.3	26.7	49.3	21.1	0.5	12.7	24.3	22.8	31.2	25.2	25.2	26.2	56.9	21.7	1.1	13.1	23.1	25.0	32.9	24.8	24.8	Dry turf	Clear
		68	4/14/23 12:45:16	24.8	40.1	26.5	0.5	11.6	26.0	24.7	27.6	23.8	23.8	26.1	49.9	22.9	0.6	12.7	24.3	23.5	31.1	24.8	24.8	26.5	61.4	23.1	0.5	13.3	25.6	24.6	34.5	25.4	25.4	Dry turf	Clear
		69	4/14/23 13:00:56	28.2	39.2	27.3	0.3	12.0	30.1	27.1	29.7	28.4	28.4	26.2	48.9	22.2	0.5	12.2	27.9	27.1	30.8	24.8	24.8	26.6	57.0	23.6	1.4	13.3	22.4	24.6	33.0	25.6	25.6	Dry turf	Clear
				**25.6**	**37.0**	**26.5**	**0.8**	**12.2**	**25.1**	**25.6**	**27.3**	**24.9**	**24.9**	**27.0**	**49.4**	**22.0**	**0.5**	**12.7**	**25.9**	**24.6**	**31.5**	**25.7**	**25.7**	**26.6**	**58.9**	**23.1**	**0.8**	**13.1**	**24.6**	**25.6**	**33.9**	**25.5**	**25.5**		
	Afternoon	70	4/14/23 15:47:31	25.2	25.9	21.3	1.4	12.1	20.9	24.9	23.3	23.4	23.4	29.6	41.7	19.4	0.7	13.1	20.9	22.8	31.1	28.5	28.5	26.7	42.0	20.7	1.3	11.2	23.3	27.1	29.0	25.2	25.2	Dry turf	Clear
		71	4/14/23 16:01:12	25.7	25.3	20.2	1.2	11.5	21.1	25.4	23.6	23.8	23.8	27.3	40.0	21.4	0.3	12.1	23.4	23.4	29.1	26.0	26.0	23.4	37.1	22.3	1.4	10.7	21.4	25.9	25.4	21.4	21.4	Dry turf	Clear
		72	4/14/23 16:16:58	25.8	22.6	24.8	0.4	12.8	24.0	25.7	23.2	24.8	24.8	28.3	36.8	22.3	0.3	13.4	23.7	24.5	28.9	27.5	27.5	27.4	30.8	18.5	1.4	12.0	21.6	26.4	26.2	25.7	25.7	Dry turf	Clear
		73	4/14/23 16:33:24	24.8	18.2	22.6	0.5	11.8	21.9	24.5	21.1	23.2	23.2	24.8	32.0	23.2	1.7	11.7	18.4	22.8	24.6	23.3	23.3	25.3	28.3	20.5	2.0	11.1	17.8	22.7	26.5	23.4	23.4	Dry turf	Clear
				**25.4**	**23.0**	**22.2**	**0.9**	**12.1**	**22.8**	**25.1**	**22.9**	**23.8**	**23.8**	**27.5**	**37.6**	**21.6**	**0.8**	**12.6**	**20.8**	**23.4**	**28.5**	**26.3**	**26.3**	**25.7**	**34.6**	**20.5**	**1.5**	**11.3**	**21.1**	**26.1**	**26.1**	**23.9**	**23.9**		
	Midday	74	4/20/23 12:19:29	22.9	33.6	32.8	3.3	11.6	20.2	23.0	21.6	22.4	22.4	23.6	47.4	34.2	0.4	13.8	25.9	22.5	29.3	23.6	23.6	25.6	48.7	31.5	0.7	13.1	24.4	23.6	30.7	25.8	25.8	Dry turf	Clear
		75	4/20/23 12:29:04	24.6	36.3	33.4	0.4	14.1	27.4	24.9	26.7	24.8	24.8	22.1	47.5	31.1	2.8	11.7	21.7	24.9	25.8	21.1	21.1	23.7	49.1	32.5	0.6	13.1	23.5	21.0	29.7	23.4	23.4	Dry turf	Clear
		76	4/20/23 12:45:12	26.3	37.2	27.8	1.2	13.0	24.4	26.4	26.2	26.0	26.0	24.5	48.0	29.1	3.3	12.7	22.1	26.2	26.9	23.9	23.9	24.9	30.9	29.1	0.4	11.3	26.4	23.9	25.1	25.4	24.3	Wet turf	Clear
		77	4/20/23 12:59:16	24.3	34.1	30.5	1.1	12.2	23.5	24.4	25.7	23.9	23.9	25.9	48.7	28.5	0.7	12.4	24.3	23.8	30.8	25.6	25.6	25.0	28.8	29.7	0.8	12.4	25.3	25.8	24.6	25.6	24.7	Wet turf	Clear
				**24.5**	**35.3**	**31.1**	**1.5**	**12.7**	**22.9**	**24.7**	**25.8**	**24.3**	**24.3**	**24.0**	**47.9**	**30.7**	**1.8**	**12.7**	**22.3**	**24.4**	**28.7**	**23.6**	**23.6**	**24.8**	**39.4**	**30.7**	**0.6**	**12.5**	**24.8**	**23.6**	**27.6**	**25.6**	**24.6**		
			**Average**	**24.7**	**30.5**	**34.3**	**1.1**	**13.8**	**23.6**	**25.0**	**24.6**	**25.0**	**25.0**	**25.3**	**43.7**	**32.4**	**1.1**	**13.8**	**23.8**	**25.2**	**28.7**	**25.5**	**25.5**	**25.4**	**46.6**	**32.6**	**1.0**	**13.9**	24.0	25.8	**29.7**	**25.7**	**25.7**		
			Maximum	**28.2**	**42.0**	**47.6**	**4.3**	**18.1**	**33.0**	**27.8**	**30.5**	**31.3**	**31.3**	**29.6**	**55.8**	**45.1**	**5.1**	**17.4**	**32.7**	**31.2**	**34.5**	**31.4**	**31.4**	**29.0**	**69.5**	**45.5**	**2.9**	**18.2**	**33.3**	**31.7**	**38.5**	**31.6**	**31.6**	**0.0**	**0.0**
			Minimum	**19.5**	**15.4**	**20.2**	**0.0**	**11.4**	**16.1**	**19.6**	**12.5**	**19.0**	**19.0**	**20.3**	**29.4**	**19.4**	**0.0**	**11.5**	**16.4**	**19.2**	**20.7**	**20.4**	**20.4**	**19.9**	**21.4**	**18.5**	**0.3**	**10.7**	**17.8**	**20.6**	**19.3**	**20.0**	**20.0**	**0.0**	**0.0**
**May**	Afternoon	78	5/3/23 14:37:04	26.7	32.2	35.0	0.8	16.1	26.2	26.4	27.1	27.9	27.9	27.2	45.0	36.2	0.7	16.3	27.1	26.8	31.0	28.9	28.9	27.0	40.5	37.0	1.5	16.0	25.1	26.7	29.3	28.8	28.8	Wet Turf	Clear
		79	5/3/23 14:53:22	27.2	29.3	38.7	0.4	16.9	28.9	26.9	26.9	29.4	29.4	26.9	41.9	38.6	0.9	15.2	26.3	26.7	30.1	29.1	29.1	27.2	44.8	39.2	0.8	16.3	26.9	27.0	31.2	29.5	29.5	Wet turf	Clear
		80	5/3/23 15:12:21	26.8	29.8	39.2	1.2	16.3	25.3	26.7	26.6	28.9	28.9	27.2	41.8	37.9	0.5	17.2	27.8	26.9	30.3	29.0	29.0	26.7	35.4	37.1	0.7	15.9	26.3	26.5	28.1	28.3	28.3	Wet turf	Clear
		81	5/3/23 15:25:23	27.9	25.7	39.5	0.6	17.3	27.8	27.5	26.5	30.6	30.6	27.9	39.4	40.4	0.8	17.2	27.1	27.6	30.3	30.8	30.8	26.5	37.5	41.3	0.4	15.8	28.1	26.6	28.8	28.9	28.9	Wet turf	Clear
				**27.2**	**29.3**	**38.1**	**0.8**	**16.7**	**26.8**	**26.9**	**26.8**	**29.2**	**29.2**	**27.3**	**42.0**	**38.3**	**0.7**	**16.5**	**27.1**	**27.0**	**30.4**	**29.4**	**29.4**	**26.9**	**39.6**	**38.7**	**0.9**	**16.0**	**26.3**	**26.7**	**29.5**	**28.9**	**28.9**		
	Afternoon	82	5/3/23 16:05:08	25.2	19.1	41.5	0.6	15.6	20.7	25.9	22.5	27.0	27.0	24.7	32.4	43.1	0.8	15.6	20.4	25.5	26.1	26.8	26.8	26.0	23.3	41.6	0.5	16.2	21.7	26.3	24.4	28.2	28.2	Wet turf	Overcast
		83	5/3/23 16:16:16	24.5	18.5	42.2	0.4	15.3	20.3	25.2	21.8	26.1	26.1	24.9	31.2	41.7	0.6	15.7	20.3	25.6	25.8	26.5	26.5	25.1	20.8	41.7	0.0	15.7	21.3	25.8	23.0	26.9	26.9	Wet turf	Overcast
		84	5/3/23 16:29:40	23.6	18.8	42.5	0.8	14.7	18.9	24.2	21.2	24.9	24.9	24.3	29.3	42.0	0.9	15.2	19.4	25.0	24.9	25.5	25.5	24.8	23.3	41.0	0.0	15.3	20.7	25.5	23.4	26.4	26.4	Wet turf	Overcast
		85	5/3/23 16:38:47	24.0	17.8	41.9	0.0	15.0	20.0	24.6	21.2	25.4	25.4	24.2	29.5	43.4	0.4	15.5	19.9	24.9	24.9	25.8	25.8	24.4	17.5	43.0	0.6	15.2	19.8	25.1	21.5	26.1	26.1	Wet turf	Overcast
				**24.3**	**18.6**	**42.0**	**0.5**	**15.2**	**20.0**	**25.0**	**21.7**	**25.9**	**25.9**	**24.5**	**30.6**	**42.6**	**0.7**	**15.5**	**19.9**	**25.3**	**25.4**	**26.1**	**26.1**	**25.1**	**21.2**	**41.8**	**0.3**	**15.6**	**21.0**	**25.8**	**23.1**	**26.9**	**26.9**		
	Midday	86	5/10/23 12:05:25	20.2	31.6	46.0	0.4	12.8	25.4	20.7	23.0	20.7	20.7	21.6	40.4	41.5	0.6	13.4	24.7	22.0	26.3	21.5	21.5	24.3	37.6	37.2	0.0	14.5	29.8	24.7	27.0	25.0	25.0	Wet turf	Clear
		87	5/10/23 12:17:00	21.7	28.0	41.5	1.1	13.6	22.7	22.1	22.6	22.1	22.1	22.5	39.3	39.8	0.8	13.3	24.0	22.9	26.5	22.7	22.7	20.0	32.3	41.9	1.6	11.9	20.4	20.2	22.1	19.9	19.9	Wet turf	Cloudy
		88	5/10/23 12:31:33	22.2	26.1	42.0	0.4	13.5	26.3	22.7	23.7	22.9	22.9	21.6	37.8	39.6	1.1	13.2	22.3	21.9	25.4	21.9	21.9	21.9	32.0	45.7	0.4	14.2	26.4	22.5	24.2	23.0	23.0	Wet turf	Cloudy
		89	5/10/23 12:44:16	21.3	17.3	43.9	0.0	13.5	28.2	21.8	19.2	21.9	21.9	21.0	31.7	42.2	0.6	12.7	24.2	21.4	23.4	21.4	21.4	19.9	22.3	42.9	0.7	12.1	22.7	20.2	19.8	19.9	19.9	Wet turf	Cloudy
				**21.4**	**25.8**	**43.4**	**0.5**	**13.4**	**25.1**	**21.8**	**21.9**	**21.9**	**21.9**	**21.7**	**37.3**	**40.8**	**0.8**	**13.2**	**23.7**	**22.0**	**25.4**	**21.9**	**21.9**	**21.5**	**31.1**	**41.9**	**0.7**	**13.2**	**24.1**	**21.9**	**23.4**	**21.9**	**21.9**		

	Afternoon	90	5/11/23 13:45:25	22.2	31.4	31.6	1.3	12.6	21.6	22.1	23.3	21.3	21.3	23.0	43.5	28.8	0.8	12.6	23.2	22.9	27.6	21.7	21.7	23.3	47.2	28.0	0.9	12.3	22.9	23.1	28.8	22.2	22.2	Dry turf	Clear
		91	5/11/23 13:56:06	23.4	31.7	26.3	2.7	11.2	20.1	23.2	22.2	22.0	22.0	22.9	42.3	28.0	0.7	11.9	23.3	22.7	27.2	21.8	21.8	22.9	41.2	28.1	2.0	11.5	20.5	22.7	25.7	21.7	21.7	Dry turf	Clear
		92	5/11/23 14:07:58	23.0	24.9	30.6	1.7	12.6	21.2	23.0	21.4	22.2	22.2	23.0	43.3	29.2	1.3	12.3	21.7	22.9	27.3	22.0	22.0	23.8	44.8	40.3	0.4	15.8	27.1	24.3	28.9	24.8	24.8	Dry turf	Clear
		93	5/11/23 14:21:21	24.3	24.7	37.5	0.9	15.6	24.5	24.7	23.3	25.1	25.1	23.5	41.5	28.1	1.9	12.7	20.9	23.4	26.3	22.7	22.7	24.3	45.7	29.9	0.6	12.7	25.0	24.3	29.0	23.8	23.8	Dry turf	Clear
				**23.2**	**28.2**	**31.5**	**1.7**	**13.0**	**21.6**	**23.2**	**22.6**	**22.6**	**22.6**	**23.1**	**42.7**	**28.5**	**1.2**	**12.4**	**22.1**	**23.0**	**27.2**	**22.0**	**22.0**	**23.6**	**44.7**	**31.6**	**1.0**	**13.1**	**23.2**	**23.6**	**28.4**	**23.1**	**23.1**		
	Afternoon	94	5/11/23 15:38:44	22.6	21.8	32.7	0.4	12.3	24.0	22.6	21.0	22.0	22.0	22.8	36.6	31.4	1.0	12.3	22.1	22.8	24.4	22.0	22.0	24.2	23.4	31.0	0.8	13.5	23.0	24.3	22.5	23.8	23.8	Dry turf	Clear
		95	5/11/23 15:47:55	23.0	20.4	31.9	0.5	13.2	23.4	23.0	20.8	22.4	22.4	22.0	35.2	30.5	0.9	12.0	20.8	21.8	24.6	21.2	21.2	21.6	22.0	33.9	2.1	11.9	19.1	21.6	19.0	20.9	20.9	Dry turf	Clear
		96	5/11/23 16:00:02	22.1	17.6	34.9	1.5	12.2	20.0	22.2	18.9	21.7	21.7	24.0	31.4	31.5	0.7	12.7	22.7	24.1	24.8	23.1	23.1	22.4	20.7	32.1	0.4	12.5	23.2	22.4	20.5	21.7	21.7	Wet turf	Clear
		97	5/11/23 16:12:31	22.2	17.6	31.4	0.6	11.6	21.6	22.1	19.4	21.3	21.3	22.8	30.4	31.4	0.3	12.3	23.8	22.8	23.7	21.9	21.9	24.0	19.7	29.8	0.5	12.1	23.2	24.0	21.2	23.4	23.4	Wet turf	Clear
				**22.5**	**19.4**	**32.7**	**0.8**	**12.3**	**21.7**	**22.5**	**20.2**	**21.9**	**21.9**	**22.9**	**33.4**	**31.2**	**0.7**	**12.3**	**22.1**	**22.9**	**24.7**	**22.1**	**22.1**	**23.1**	**21.5**	**31.7**	**1.0**	**12.5**	**21.6**	**23.1**	**21.2**	**22.4**	**22.4**		
	Midday	98	5/19/23 10:50:29	17.5	25.0	59.4	1.5	12.8	20.0	18.4	19.0	18.5	18.5	17.2	30.9	57.0	0.6	11.8	22.4	18.0	21.4	18.0	18.0	17.6	29.4	64.0	0.7	14.6	22.6	18.8	21.5	19.2	19.2	Wet turf	Clear
		99	5/19/23 10:59:53	19.4	24.5	49.5	0.0	12.9	27.3	20.0	20.4	20.0	20.0	17.7	31.5	50.1	1.2	11.2	20.1	18.1	21.4	18.4	18.4	19.3	29.1	56.6	0.4	14.2	25.4	20.2	22.1	20.8	20.8	Wet turf	Clear
		100	5/19/23 11:14:38	20.1	21.8	47.4	0.6	12.8	24.1	20.6	19.9	20.7	20.7	17.4	33.0	49.1	1.9	10.5	18.6	17.8	20.4	18.3	18.3	18.8	27.8	52.7	0.8	13.1	22.4	19.5	21.2	19.6	19.6	Wet turf	Clear
		101	5/19/23 11:25:49	18.7	23.7	48.4	0.9	11.2	21.7	19.2	19.7	18.9	18.9	18.5	34.9	47.6	1.7	12.2	19.7	18.9	22.0	18.6	18.6	18.6	29.0	49.3	0.5	12.3	23.7	19.1	21.4	18.9	18.9	Wet turf	Clear
				**18.9**	**23.8**	**51.2**	**0.8**	**12.4**	**22.6**	**19.5**	**19.9**	**19.6**	**19.6**	**17.7**	**32.6**	**51.0**	**1.4**	**11.4**	**19.9**	**18.2**	**20.9**	**18.3**	**18.3**	**18.6**	**28.8**	**55.7**	**0.6**	**13.6**	**23.5**	**19.4**	**21.6**	**19.6**	**19.6**		
	Afternoon	102	5/22/23 13:43:11	20.2	26.9	25.9	0.7	9.8	21.3	19.6	20.9	18.0	18.0	20.8	42.0	24.5	0.7	9.4	21.5	20.2	25.9	18.5	18.5	21.6	47.5	24.1	0.3	9.9	25.1	21.1	27.9	19.5	19.5	Dry turf	Clear
		103	5/22/23 13:59:34	22.2	25.6	26.0	0.6	11.3	23.3	21.8	21.7	20.5	20.5	22.1	41.1	25.7	0.5	11.3	23.8	21.7	26.3	20.4	20.4	22.3	42.2	24.6	0.4	11.0	24.5	21.9	26.7	20.4	20.4	Dry turf	Clear
		104	5/22/23 14:13:48	21.8	24.1	25.5	0.4	10.2	24.2	21.4	21.0	19.9	19.9	21.9	40.8	25.4	1.2	9.9	20.7	21.5	25.9	20.0	20.0	22.4	41.6	26.6	0.0	11.5	27.1	22.1	26.7	20.8	20.8	Dry turf	Clear
		105	5/22/23 14:18:19	20.4	24.2	27.9	0.4	9.9	23.4	20.0	20.2	18.6	18.6	20.9	38.3	25.2	1.0	9.9	20.4	20.4	24.8	18.7	18.7	21.5	37.1	31.6	1.1	12.2	21.2	21.4	24.8	20.4	20.4	Dry turf	Clear
				**21.2**	**25.2**	**26.3**	**0.5**	**10.3**	**22.8**	**20.7**	**20.9**	**19.3**	**19.3**	**21.4**	**40.6**	**25.2**	**0.9**	**10.1**	**21.4**	**20.9**	**25.8**	**19.4**	**19.4**	**22.0**	**42.1**	**26.7**	**0.5**	**11.2**	**24.1**	**21.6**	**26.6**	**20.3**	**20.3**		
	Afternoon	106	5/22/23 14:44:23	21.1	21.5	28.0	0.5	10.5	22.8	20.7	19.8	19.4	19.4	21.8	38.1	25.1	0.8	10.3	21.4	21.3	25.3	19.7	19.7	22.2	36.8	28.2	0.5	11.6	23.7	21.9	25.1	20.8	20.8	Dry turf	Clear
		107	5/22/23 14:57:09	22.1	22.6	27.5	0.3	11.1	24.9	21.8	20.7	20.6	20.6	22.5	37.2	26.1	1.2	11.3	20.8	22.2	25.2	20.8	20.8	21.8	35.0	25.2	0.8	10.9	21.3	21.4	24.3	19.9	19.9	Dry turf	Clear
		108	5/22/23 15:12:34	21.7	22.1	26.8	0.4	10.9	23.4	21.3	20.3	20.0	20.0	21.3	33.1	25.0	0.7	10.0	21.0	20.8	23.5	19.3	19.3	22.3	33.8	27.1	0.6	11.2	22.6	22.0	24.3	20.8	20.8	Dry turf	Clear
		109	5/23/23 15:26:56	21.5	22.9	27.2	0.4	10.7	23.0	21.1	20.4	19.8	19.8	21.8	37.2	25.4	0.9	10.4	20.6	21.4	25.0	19.9	19.9	22.1	33.8	26.8	0.6	11.2	22.2	21.8	24.2	20.5	20.5	Dry turf	Clear
				**21.5**	**22.9**	**27.2**	**0.4**	**10.7**	**23.1**	**21.1**	**20.4**	**19.8**	**19.8**	**21.8**	**37.2**	**25.4**	**0.9**	**10.4**	**20.8**	**21.3**	**25.0**	**19.8**	**19.8**	**22.1**	**36.9**	**26.8**	**0.6**	**11.2**	**22.5**	**21.7**	**25.1**	**20.5**	**20.5**		
			**Average**	**22.5**	**24.1**	**36.6**	**0.7**	**13.0**	**23.3**	**22.7**	**21.8**	**22.5**	**22.5**	**22.6**	**37.0**	**35.4**	**0.9**	**12.7**	**22.2**	**22.7**	**25.6**	**22.4**	**22.4**	**22.8**	**33.0**	**36.9**	**0.7**	**13.3**	**23.5**	**23.1**	**24.7**	**23.0**	**23.0**		
			**Maximum**	**27.9**	**32.2**	**59.4**	**2.7**	**17.3**	**28.9**	**27.5**	**27.1**	**30.6**	**30.6**	**27.9**	**45.0**	**57.0**	**1.9**	**17.2**	**27.8**	**27.6**	**31.0**	**30.8**	**30.8**	**27.2**	**47.5**	**64.0**	**2.1**	**16.3**	**29.8**	**27.0**	**31.2**	**29.5**	**29.5**		
			**Minimum**	**17.5**	**17.3**	**25.5**	**0.0**	**9.8**	**18.9**	**18.4**	**18.9**	**18.0**	**18.0**	**17.2**	**29.3**	**24.5**	**0.3**	**9.4**	**18.6**	**17.8**	**20.4**	**18.0**	**18.0**	**17.6**	**17.5**	**24.1**	**0.0**	**9.9**	**19.1**	**18.8**	**19.0**	**18.9**	**18.9**		
	Midday	109	6/6/23 10:54:39	18.3	23.4	51.7	1.9	12.6	19.4	18.9	18.0	18.8	18.8	19.7	31.5	49.5	1.5	12.8	21.0	20.3	22.1	20.4	20.4	19.3	28.6	52.3	0.6	13.2	23.7	20.0	21.8	20.2	20.2	Wet turf	Clear
		110	6/6/23 11:07:42	18.6	25.5	51.3	0.8	12.3	22.1	19.2	20.3	19.1	19.1	18.2	30.5	52.6	2.0	12.6	19.4	18.8	20.0	18.7	18.7	18.2	30.5	54.5	1.0	13.1	21.4	18.9	21.8	19.0	19.0	Wet turf	Clear
		111	6/6/23 11:17:13	18.8	26.2	47.2	0.9	13.0	21.9	19.2	20.5	18.9	18.9	17.3	32.2	52.1	1.1	11.8	20.1	17.8	21.6	17.5	17.5	19.5	35.0	51.4	1.9	12.8	20.5	20.2	25.8	20.4	20.4	Wet turf	Clear
		112	6/6/23 11:36:10	19.1	24.9	48.1	0.4	12.3	24.7	19.6	20.3	19.4	19.4	18.9	32.9	50.0	1.6	12.5	20.3	19.5	21.9	19.4	19.4	19.0	30.3	50.9	0.9	13.3	22.1	19.6	22.1	19.6	19.6	Wet turf	Clear
				**18.7**	**25.0**	**49.6**	**1.0**	**12.6**	**21.4**	**19.2**	**20.1**	**19.1**	**19.1**	**18.5**	**31.8**	**51.1**	**1.6**	**12.4**	**20.1**	**19.1**	**21.3**	**19.0**	**19.0**	**19.0**	**31.1**	**52.3**	**1.1**	**13.1**	**21.5**	**19.7**	**22.3**	**19.8**	**19.8**		
	Afternoon	113	6/6/23 13:52:42	19.5	26.4	46.3	3.3	12.7	18.8	19.9	17.1	19.8	19.8	20.3	39.2	44.5	2.0	12.7	20.2	20.7	23.8	20.6	20.6	21.1	39.7	44.4	0.8	13.5	23.3	21.6	25.9	21.7	21.7	Dry turf	Clear
		114	6/6/23 14:02:23	20.9	27.0	45.5	0.7	13.4	23.6	21.4	22.0	21.6	21.6	21.4	36.4	43.4	2.2	13.7	20.8	21.9	23.4	22.0	22.0	21.6	39.0	44.6	0.4	14.2	25.9	22.1	26.0	22.4	22.4	Dry turf	Clear
		115	6/6/23 14:16:14	21	25.0	44.0	0.5	13.2	24.6	21.5	21.4	21.5	21.5	22.2	34.6	45.1	0.7	14.1	24.4	22.8	25.1	23.3	23.3	21.4	37.5	42.2	0.6	13.4	24.2	21.8	25.4	21.8	21.8	Dry turf	Clear
		116	6/6/23 14:35:16	21.1	23.8	45.5	1.0	14.7	22.6	21.6	21.1	21.9	21.9	21.0	34.2	42.4	1.0	13.1	22.2	21.4	24.2	21.3	21.3	20.5	34.5	42.3	0.8	12.5	22.3	20.8	23.9	20.6	20.6	Wet turf	Clear
				**20.6**	**25.6**	**45.3**	**1.4**	**13.5**	**21.4**	**21.1**	**20.1**	**21.2**	**21.2**	**21.2**	**36.1**	**43.9**	**1.5**	**13.4**	**21.6**	**21.7**	**24.3**	**21.8**	**21.8**	**21.2**	**37.7**	**43.4**	**0.7**	**13.4**	**23.7**	**21.6**	**25.4**	**21.6**	**21.6**		
	Midday	117	6/9/23 11:43:28	18.2	28.7	27.5	0.7	8.1	20.5	17.5	20.4	15.8	15.8	19.6	35.7	25.4	0.7	8.8	20.9	18.9	23.3	17.3	17.3	19.8	38.8	25.5	0.5	8.6	22.4	19.2	24.4	17.5	17.5	Dry turf	Clear
		118	6/9/23 11:59:38	18.5	25.9	22.5	1.8	7.6	17.1	17.6	18.3	15.6	15.6	20.3	36.2	22.8	0.6	9.0	21.8	19.6	23.8	17.8	17.8	18.4	39.8	23.8	1.6	8.3	17.4	17.5	23.0	15.6	15.6	Dry turf	Clear
		119	6/9/23 12:15:59	20.2	29.6	26.4	0.6	9.5	22.1	19.7	21.8	18.1	18.1	20.2	36.8	23.4	1.2	9.1	19.4	19.5	23.7	17.7	17.7	19.9	38.7	22.3	1.8	8.6	18.1	19.1	23.2	17.2	17.2	Dry turf	Clear
		120	6/9/23 12:36:05	21.4	29.7	24.8	1.0	10.3	21.0	20.9	22.4	19.4	19.4	19.3	37.9	23.2	1.3	9.0	18.6	18.5	23.4	16.6	16.6	20.9	40.6	23.7	0.5	10.0	22.8	20.3	25.5	18.6	18.6	Dry turf	Clear
				**19.6**	**28.5**	**25.3**	**1.0**	**8.9**	**19.7**	**18.9**	**21.0**	**17.2**	**17.2**	**19.9**	**36.7**	**23.7**	**1.0**	**9.0**	**20.0**	**19.1**	**23.8**	**17.3**	**17.3**	**19.8**	**39.5**	**23.8**	**1.1**	**8.9**	**19.4**	**19.0**	**24.5**	**17.2**	**17.2**		
	Midday	121	6/17/23 10:39:52	18.2	23.3	34.1	0.6	9.1	21.3	17.9	18.8	16.6	16.6	17.0	31.2	33.8	1.6	8.6	17.2	16.5	19.6	15.1	15.1	18.2	41.6	30.6	2.4	8.6	16.9	17.7	22.1	16.2	16.2	Dry turf	Clear
		122	6/17/23 10:56:49	19.4	22.3	31.1	0.8	9.7	20.8	19.0	19.1	17.7	17.7	17.7	27.3	31.5	1.7	8.8	17.5	17.2	18.6	15.7	15.7	21.3	41.2	27.9	1.3	10.4	20.4	20.9	25.6	19.7	19.7	Dry turf	Clear
		123	6/17/23 11:10:39	21.2	22.5	27.9	0.8	9.5	21.9	20.8	20.1	19.5	19.5	18.4	30.2	31.9	1.3	8.8	18.8	18.0	20.6	16.6	16.6	21.3	44.1	28.2	1.3	10.7	20.6	21.0	26.5	19.7	19.7	Dry turf	Clear
June		124	6/17/23 11:26:19	21.2	21.4	28.7	1.3	10.6	20.6	20.9	19.5	19.7	19.7	18.7	28.7	32.3	1.3	9.8	19.1	18.3	20.3	17.0	17.0	20.2	43.3	29.7	1.3	10.3	19.9	19.8	25.7	18.5	18.5	Dry turf	Clear
				**20.0**	**22.4**	**30.5**	**0.9**	**9.7**	**20.9**	**19.6**	**19.4**	**18.4**	**18.4**	**18.0**	**29.4**	**32.4**	**1.5**	**9.0**	**18.1**	**17.5**	**19.8**	**16.1**	**16.1**	**20.3**	**42.6**	**29.1**	**1.6**	**10.0**	**19.4**	**19.9**	**27.4**	**18.5**	**18.5**		
	Morning	125	6/19/23 9:26:06	17	15.2	34.2	0.4	9.1	21.1	16.5	15.3	15.1	15.1	16.4	19.5	34.3	0.9	7.9	17.9	15.9	16.4	14.4	14.4	16.7	21.5	34.7	1.0	8.6	17.9	16.2	17.3	14.8	14.8	Wet turf	Clear
		126	6/19/23 9:36:29	16.4	15.9	31.0	0.9	7.8	17.8	15.7	15.1	14.1	14.1	16.2	20.3	32.1	1.7	7.6	16.0	15.6	15.3	13.9	13.9	17.6	21.1	32.5	0.6	9.2	20.2	17.1	17.6	15.7	15.7	Wet turf	Clear
		127	6/19/23 9:44:05	18	16.7	30.1	0.6	8.5	20.3	17.4	16.3	15.9	15.9	17.5	19.9	30.1	0.7	8.5	19.3	16.9	17.1	15.3	15.3	17.8	21.2	30.9	0.6	8.7	20.2	17.2	17.7	15.7	15.7	Wet turf	Clear
		128	6/19/23 9:54:42	18.1	17.4	30.8	0.4	8.6	21.9	17.6	16.7	16.1	16.1	17.2	21.8	30.3	1.4	8.8	17.2	16.6	16.9	14.9	14.9	18.3	21.8	30.5	0.8	9.3	19.6	17.8	18.2	16.3	16.3	Wet turf	Clear
				**17.4**	**16.3**	**31.5**	**0.6**	**8.5**	**20.0**	**16.8**	**15.9**	**15.3**	**15.3**	**16.8**	**20.4**	**31.7**	**1.2**	**8.2**	**17.4**	**16.2**	**16.6**	**14.6**	**14.6**	**17.6**	**21.4**	**32.2**	**0.8**	**9.0**	**19.3**	**17.1**	**17.7**	**15.6**	**15.6**		
	Midday	129	6/19/23 12:07:07	20.2	28.5	29.7	0.6	10.3	22.4	19.8	21.5	18.5	18.5	19.6	35.2	28.4	2.4	9.7	17.8	19.1	21.0	17.6	17.6	21.3	37.4	27.0	1.7	10.3	19.7	20.9	23.9	19.5	19.5	Dry turf	Clear
		130	6/19/23 12:16:20	21.2	28.3	26.6	1.4	10.4	20.2	20.8	21.5	19.4	19.4	21.0	34.2	27.2	1.8	10.3	19.4	20.6	22.6	19.2	19.2	21.2	35.5	28.1	1.3	10.7	20.5	20.8	23.9	19.6	19.6	Dry turf	Clear
		131	6/19/23 12:30:55	22.1	29.3	26.6	1.3	11.2	20.9	21.8	22.5	20.5	20.5	20.6	34.1	26.1	2.4	10.0	18.3	20.1	21.3	18.6	18.6	21.5	34.6	26.6	2.3	10.2	19.1	21.1	22.2	19.7	19.7	Dry turf	Clear
		132	6/19/23 12:42:47	22.5	26.3	25.1	0.8	10.6	22.4	22.1	22.1	20.7	20.7	21.7	34.4	25.3	2.1	9.8	19.3	21.2	22.6	19.8	19.8	22.4	35.3	26.5	1.5	11.1	20.7	22.1	24.2	20.8	20.8	Dry turf	Clear
				**21.5**	**28.1**	**27.0**	**1.0**	**10.6**	**21.2**	**21.1**	**22.1**	**19.8**	**19.8**	**20.7**	**34.5**	**26.8**	**2.2**	**10.0**	**18.7**	**20.3**	**21.8**	**18.8**	**18.8**	**21.6**	**35.7**	**27.1**	**1.7**	**10.6**	**19.9**	**21.2**	**23.6**	**19.9**	**19.9**		
	Midday	133	6/22/23 12:05:30	21.2	28.2	22.4	0.5	9.4	23.0	20.5	21.8	18.8	18.8	21.3	36.5	22.1	0.9	9.7	21.0	20.6	24.4	18.8	18.8	21.4	45.7	26.2	0.4	10.1	24.3	21.0	27.3	19.5	19.5	Dry turf	Clear
		134	6/22/23 12:18:00	21.1	31.2	22.9	0.3	9.6	24.7	20.5	22.7	18.7	18.7	20.4	36.2	22.9	0.8	9.3	20.7	19.7	23.9	17.9	17.9	20.1	46.2	22.5	0.5	9.0	22.2	19.3	26.8	17.5	17.5	Dry turf	Clear
		135	6/22/23 12:30:32	21	30.3	21.6	0.4	9.5	23.4	20.3	22.3	18.4	18.4	19.9	35.4	21.1	1.0	8.8	19.6	19.0	23.3	17.1	17.1	21.7	46.6	21.3	0.8	9.2	21.4	21.0	27.7	19.2	19.2	Dry turf	Clear
		136	6/22/23 12:45:53	21.4	31.2	24.2	1.0	10.2	20.9	20.9	22.9	19.3	19.3	21.0	35.7	22.2	0.5	9.9	22.7	20.3	24.0	18.5	18.5	20.8	44.2	21.7	1.7	9.5	18.7	20.1	25.6	18.2	18.2	Dry turf	Clear
				**21.2**	**30.2**	**22.8**	**0.6**	**9.7**	**22.7**	**20.5**	**22.4**	**18.8**	**18.8**	**20.7**	**36.0**	**22.1**	**0.8**	**9.4**	**20.8**	**19.9**	**24.0**	**18.1**	**18.1**	**21.0**	**45.7**	**22.9**	**0.9**	**9.5**	**21.0**	**20.4**	**27.1**	**18.6**	**18.6**		
	After[illegible]	137	6/22/23 13:52:12	20.4	27.1	22.5	0.3	9.2	24.1	19.7	21.0	17.8	17.8	21.1	38.1	21.3	0.8	9.3	20.9	20.4	24.8	18.5	18.5	20.2	23.3	26.5	1.2	9.9	19.7	19.7	19.5	18.1	18.1	Wet turf	Clear
		138	6/22/23 14:06:49	21.8	27.1	23.1	0.8	10.3	21.6	21.2	21.8	19.6	19.6	22.0	35.7	20.4	0.5	10.8	23.1	21.3	24.6	19.4	19.4	20.8	25.1	23.5	0.7	9.8	21.3	20.2	20.6	18.4	18.4	Wet turf	Clear

July

	139	6/22/23 14:19:35	20.6	26.4	22.7	0.0	9.5	25.4	19.9	20.9	18.1	18.1	21.9	34.7	22.9	0.4	10.4	23.9	21.3	24.2	19.7	19.7	21.8	26.2	23.6	0.4	10.5	23.9	21.3	21.6	19.7	19.7	Wet turf	Clear
	140	6/22/23 14:36:48	21.1	22.7	23.3	0.4	9.9	23.3	20.5	20.0	18.8	18.8	21.1	32.8	23.0	0.9	9.8	20.5	20.5	23.2	18.7	18.7	21.8	23.8	22.5	0.0	10.3	26.0	21.2	20.8	19.5	19.5	Wet turf	Clear
			**21.0**	**25.8**	**22.9**	**0.4**	**9.7**	**23.8**	**20.3**	**20.9**	**18.6**	**18.6**	**21.5**	**35.3**	**21.9**	**0.7**	**10.1**	**21.8**	**20.9**	**24.2**	**19.1**	**19.1**	**21.2**	**24.6**	**24.0**	**0.6**	**10.1**	**22.2**	**20.6**	**22.5**	**18.9**	**18.9**		
		**Average**	**20.0**	**25.2**	**31.9**	**0.9**	**10.4**	**21.7**	**19.7**	**20.2**	**18.5**	**18.5**	**19.7**	**32.5**	**31.7**	**1.3**	**10.2**	**20.0**	**19.3**	**22.0**	**18.1**	**18.1**	**20.2**	**34.8**	**31.8**	**1.0**	**10.6**	**21.1**	**19.9**	**23.8**	**18.8**	**18.8**		
		**Maximum**	**22.5**	**31.2**	**51.7**	**3.3**	**14.7**	**25.4**	**22.1**	**22.9**	**21.9**	**21.9**	**22.2**	**39.2**	**52.6**	**2.4**	**14.1**	**24.4**	**22.8**	**25.1**	**23.3**	**23.3**	**22.4**	**46.6**	**54.5**	**2.4**	**14.2**	**26.0**	**22.1**	**27.7**	**22.4**	**22.4**		
		**Minimum**	**16.4**	**15.2**	**21.6**	**0.0**	**7.6**	**17.1**	**15.7**	**15.1**	**14.1**	**14.1**	**16.2**	**19.5**	**20.4**	**0.4**	**7.6**	**16.0**	**15.6**	**15.3**	**13.9**	**13.9**	**16.7**	**21.1**	**21.3**	**0.0**	**8.3**	**16.9**	**16.2**	**17.3**	**14.8**	**14.8**		
Midday	141	7/1/23 10:32:58	16.4	24.1	31.9	1.1	8.0	18.2	15.8	17.8	14.1	14.1	16.4	27.0	29.5	1.2	7.3	17.1	15.6	18.5	13.9	13.9	17.4	41.8	34.8	0.8	9.2	19.6	17.0	24.3	15.7	15.7	Dry turf	Clear
	142	7/1/23 10:47:24	16.7	25.5	29.4	0.6	8.3	19.8	16.0	18.5	14.2	14.2	17.3	28.1	27.9	0.8	8.3	18.9	16.5	19.7	14.8	14.8	18.2	43.4	31.2	0.4	9.2	22.4	17.7	25.1	16.3	16.3	Dry turf	Clear
	143	7/1/23 10:59:56	17.8	25.3	32.4	0.4	9.4	22.3	17.3	19.1	15.9	15.9	16.9	28.7	29.2	0.7	8.1	19.2	16.2	19.7	14.5	14.5	18.1	43.1	29.9	0.7	9.0	20.2	17.5	24.9	16.0	16.0	Dry turf	Clear
	144	7/1/23 11:21:44	18.6	25.9	27.6	0.4	9.0	22.5	18.0	19.7	16.3	16.3	18.5	31.2	26.9	1.1	8.6	18.8	17.8	21.2	16.1	16.1	19.0	45.4	27.1	0.4	8.9	22.7	18.4	26.0	16.7	16.7	Dry turf	Clear
			**17.4**	**25.2**	**30.3**	**0.6**	**8.7**	**20.1**	**16.8**	**18.8**	**15.2**	**15.2**	**17.3**	**28.8**	**28.4**	**1.0**	**8.1**	**18.5**	**16.5**	**19.9**	**14.8**	**14.8**	**18.2**	**43.4**	**30.8**	**0.6**	**9.1**	**20.9**	**17.7**	**25.1**	**16.2**	**16.2**		
Afternoon	145	7/2/23 14:54:49	21.5	25.1	31.6	0.4	11.5	24.2	21.4	21.3	20.4	20.4	22.5	33.6	27.8	0.0	11.4	26.7	22.3	24.4	21.1	21.1	20.9	36.2	27.6	0.7	9.9	21.4	20.5	24.2	19.1	19.1	Dry turf	Clear
	146	7/2/23 15:12:43	20.9	21.4	28.2	0.5	10.6	22.4	20.5	19.6	19.2	19.2	19.8	29.8	27.8	1.4	9.8	18.6	19.3	21.1	17.8	17.8	20.9	32.4	27.3	0.7	10.1	21.1	20.5	23.1	19.1	19.1	Dry turf	Clear
	147	7/2/23 15:29:48	20.4	19.4	28.3	0.9	9.9	19.9	20.0	18.7	18.6	18.6	20.8	28.0	27.9	0.4	10.5	22.7	20.4	21.6	19.0	19.0	20.7	29.2	27.3	0.6	10.3	21.3	20.3	21.9	18.8	18.8	Dry turf	Clear
	148	7/2/23 15:42:51	20.9	16.6	27.2	0.4	9.8	22.4	20.5	18.0	19.1	19.1	19.3	25.5	28.0	1.7	9.4	17.3	18.7	18.9	17.2	17.2	20.4	16.8	28.9	0.7	10.2	20.4	20.0	17.8	18.7	18.7	Wet turf	Clear
			**20.9**	**20.6**	**28.8**	**0.6**	**10.5**	**22.1**	**20.6**	**19.4**	**19.3**	**19.3**	**20.6**	**29.2**	**27.9**	**0.9**	**10.3**	**20.2**	**20.2**	**21.9**	**18.8**	**18.8**	**20.7**	**28.7**	**27.8**	**0.7**	**10.1**	**21.1**	**20.3**	**21.8**	**18.9**	**18.9**		
Midday	149	7/3/23 11:22:40	17.6	32.7	44.1	1.1	10.6	19.7	17.7	21.6	17.0	17.0	17.8	31.3	37.1	0.3	9.8	23.7	17.6	21.2	16.4	16.4	18.7	44.0	35.7	0.8	10.0	20.8	18.5	25.6	17.4	17.4	Dry turf	Clear
	150	7/3/23 11:28:13	19.6	30.3	34.4	0.0	10.6	26.3	19.4	21.8	18.4	18.4	17.9	32.7	34.5	0.6	9.8	21.2	17.5	21.7	16.3	16.3	19.1	46.0	35.1	0.4	10.0	23.5	18.9	26.4	17.9	17.9	Dry turf	Clear
	151	7/3/23 11:39:55	20.5	32.1	34.7	0.4	11.5	24.5	20.4	22.9	19.6	19.6	18.4	33.1	32.8	0.7	10.3	20.7	18.0	22.0	16.7	16.7	20.5	46.4	32.7	1.0	12.1	21.3	20.3	27.2	19.3	19.3	Dry turf	Clear
	152	7/3/23 11:47:42	18.0	25.1	37.9	0.4	10.6	23.0	17.8	19.3	16.8	16.8	19.0	33.0	35.1	0.4	9.9	23.4	18.8	22.4	17.7	17.7	20.1	45.3	34.8	0.8	11.5	21.8	20.0	26.7	19.1	19.1	Dry turf	Clear
			**18.9**	**30.1**	**37.8**	**0.5**	**10.8**	**22.9**	**18.8**	**21.5**	**18.0**	**18.0**	**18.3**	**32.5**	**34.9**	**0.5**	**10.0**	**22.2**	**18.0**	**21.8**	**16.8**	**16.8**	**19.6**	**45.4**	**34.6**	**0.8**	**10.9**	**21.6**	**19.4**	**26.5**	**18.4**	**18.4**		
Afternoon	153	7/3/23 13:53:07	19.2	26.7	32.2	0.4	9.9	23.2	18.9	20.4	17.6	17.6	20.3	35.8	30.6	0.6	10.5	22.4	20.0	23.9	18.8	18.8	21.3	46.2	26.5	0.0	10.5	26.5	20.9	27.4	19.5	19.5	Dry turf	Clear
	154	7/3/23 14:10:24	20.7	27.8	29.9	0.0	11.0	26.3	20.4	21.6	19.2	19.2	20.7	35.5	28.7	0.4	10.2	23.8	20.3	23.9	19.0	19.0	21.2	44.3	27.3	0.0	10.7	26.4	20.8	26.8	19.5	19.5	Dry turf	Clear
	155	7/3/23 14:17:37	19.7	24.4	28.5	1.0	9.8	20.0	19.2	19.9	17.8	17.8	20.1	33.8	28.1	1.0	9.8	20.2	19.6	23.1	18.2	18.2	21.4	41.7	30.2	0.5	10.7	23.6	21.2	26.2	20.1	20.1	Dry turf	Clear
	156	7/3/23 14:23:09	20.0	25.8	28.9	0.9	11.3	20.5	19.6	20.5	18.2	18.2	19.8	33.2	29.3	0.7	9.9	21.1	19.4	22.7	18.0	18.0	20.9	41.3	30.4	0.4	11.0	23.9	20.6	25.8	19.5	19.5	Dry turf	Clear
			**19.9**	**26.2**	**29.9**	**0.6**	**10.5**	**21.9**	**19.5**	**20.6**	**18.2**	**18.2**	**20.2**	**34.6**	**29.2**	**0.7**	**10.1**	**21.7**	**19.8**	**23.4**	**18.5**	**18.5**	**21.2**	**43.4**	**28.6**	**0.2**	**10.7**	**26.5**	**20.9**	**26.6**	**19.6**	**19.6**		
Midday	157	7/8/23 13:03:24	17.8	27.1	37.3	1.4	10.0	18.6	17.6	19.2	16.5	16.5	20.3	37.8	34.4	0.5	11.1	23.5	20.2	24.6	19.3	19.3	19.6	39.6	35.5	1.3	11.0	20.0	19.5	24.4	18.5	18.5	Dry turf	Clear
	158	7/8/23 13:26:21	19.0	28.3	34.0	0.6	9.9	21.9	18.7	20.8	17.6	17.6	20.3	36.6	33.0	0.8	10.2	21.7	20.1	24.2	19.1	19.1	20.1	38.4	33.8	0.8	11.0	20.0	19.9	24.6	19.0	19.0	Dry turf	Clear
	159	7/8/23 13:44:53	20.9	26.3	33.5	0.7	11.5	22.5	20.8	21.3	19.9	19.9	20.3	35.5	30.1	0.5	10.5	23.0	20.0	23.7	18.7	18.7	21.1	25.5	32.3	0.5	11.6	23.8	21.0	21.2	20.0	20.0	Wet turf	Clear
	160	7/8/23 14:04:47	19.6	26.1	32.5	0.4	10.7	23.4	19.3	20.4	18.2	18.2	19.9	35.0	30.4	0.8	10.0	21.0	19.5	23.4	18.3	18.3	20.3	23.5	31.5	1.4	10.7	19.8	20.0	19.5	18.9	18.9	Wet turf	Clear
			**19.3**	**27.0**	**34.3**	**0.8**	**10.5**	**21.3**	**19.1**	**20.6**	**18.0**	**18.0**	**20.2**	**36.2**	**32.0**	**0.7**	**10.5**	**22.1**	**19.9**	**24.0**	**18.8**	**18.8**	**20.3**	**31.8**	**33.3**	**1.0**	**11.1**	**21.1**	**20.1**	**22.7**	**19.1**	**19.1**		
Midday	169	7/15/23 11:56:13	13.2	26.4	52.8	0.6	8.8	19.1	13.3	17.5	12.1	12.1	12.7	29.0	52.6	1.9	7.9	15.0	12.8	16.1	11.4	11.4	12.8	25.1	54.0	0.7	8.2	18.1	13.0	16.8	11.7	11.7	Wet turf	Clear
	170	7/15/23 12:12:10	14.6	27.1	53.7	0.6	7.9	20.2	14.9	20.0	14.0	14.0	15.3	29.7	51.4	0.5	10.3	21.2	15.6	19.7	14.7	14.7	15.2	25.2	53.4	1.0	9.8	18.9	15.6	18.2	14.8	14.8	Wet turf	Clear
	171	7/15/23 12:37:15	12.5	24.3	50.3	1.9	7.2	14.6	12.4	14.3	11.0	11.0	15.2	31.8	49.2	0.5	9.0	20.9	15.3	20.3	14.4	14.4	13.9	24.6	49.4	0.6	7.5	19.3	13.9	17.2	12.7	12.7	Wet turf	Clear
	172	7/15/23 12:51:38	13.9	16.7	46.9	0.8	7.8	18.0	13.8	14.3	12.5	12.5	13.3	28.5	48.2	1.5	7.8	15.7	13.2	17.1	11.8	11.8	13.2	17.0	50.9	0.8	7.6	17.8	13.2	14.2	11.9	11.9	Wet turf	Overcast
			**13.6**	**23.6**	**50.9**	**1.0**	**7.9**	**17.4**	**13.6**	**16.7**	**12.4**	**12.4**	**14.1**	**29.8**	**50.4**	**1.1**	**8.8**	**17.4**	**14.2**	**18.9**	**13.1**	**13.1**	**13.8**	**23.0**	**51.9**	**0.8**	**8.3**	**18.5**	**13.9**	**16.6**	**12.8**	**12.8**		
Midday	173	7/15/23 13:39:02	12.5	12.0	50.1	1.6	7.2	15.0	12.4	10.7	11.0	11.0	13.0	29.1	49.8	0.9	7.7	17.1	13.0	18.2	11.6	11.6	12.4	12.1	51.0	0.4	7.1	19.7	12.4	11.9	10.9	10.9	Wet turf	Overcast
	174	7/15/23 13:55:34	12.3	13.6	50.9	0.7	7.1	17.3	12.2	12.4	10.8	10.8	12.5	22.1	50.8	0.7	7.8	17.5	12.5	15.5	11.0	11.0	12.9	11.8	50.5	0.4	7.5	19.9	12.9	12.1	11.5	11.5	Wet turf	Overcast
	175	7/15/23 14:10:45	12.4	13.4	49.8	0.7	7.1	17.2	12.3	12.4	10.8	10.8	13.0	21.1	49.1	0.4	7.1	19.8	12.9	15.4	11.5	11.5	12.8	13.3	50.0	0.6	7.3	18.3	12.7	12.6	11.3	11.3	Wet turf	Overcast
	176	7/15/23 14:26:16	13.5	13.6	42.7	0.8	7.6	17.1	13.1	12.9	11.6	11.6	14.8	20.5	44.0	0.5	7.7	19.9	14.6	16.1	13.4	13.4	13.4	13.2	41.9	0.5	7.6	18.7	13.0	12.7	11.4	11.4	Wet turf	Overcast
			**12.7**	**13.2**	**48.4**	**1.0**	**7.3**	**16.5**	**12.5**	**12.4**	**11.1**	**11.1**	**13.3**	**23.2**	**48.4**	**0.6**	**7.6**	**18.4**	**13.2**	**16.3**	**11.9**	**11.9**	**12.9**	**12.6**	**48.4**	**0.5**	**7.4**	**18.9**	**12.7**	**12.3**	**11.3**	**11.3**		
Midday	177	7/19/23 12:08:04	20.1	32.5	26.5	0.4	9.6	23.4	19.5	22.6	18.0	18.0	21.7	38.1	20.7	0.5	9.7	23.2	21.0	25.1	19.1	19.1	21.1	33.2	24.5	0.9	9.4	21.1	20.5	23.4	18.9	18.9	Wet turf	Clear
	178	7/19/23 12:22:14	19.3	25.0	21.3	1.3	8.1	18.4	18.4	19.3	16.4	16.4	21.5	37.3	21.5	0.4	9.5	23.8	20.8	24.8	19.0	19.0	20.2	25.4	22.8	1.5	8.4	18.8	19.5	19.7	17.6	17.6	Wet turf	Clear
	179	7/19/23 12:30:46	19.8	29.8	19.7	2.6	8.2	16.8	18.9	18.9	16.8	16.8	21.0	36.2	20.1	3.9	9.2	16.9	20.2	19.5	18.2	18.2	20.4	25.3	21.5	3.3	8.4	16.9	19.6	16.7	17.7	17.7	Wet turf	Clear
	180	7/19/23 12:42:41	21.3	29.4	20.6	1.1	9.0	20.1	20.6	22.1	18.6	18.6	20.8	38.2	20.6	0.6	9.4	21.8	20.0	24.7	18.1	18.1	20.7	27.3	27.1	0.9	11.6	21.1	20.2	21.3	18.8	18.8	Wet turf	Clear
			**20.1**	**29.2**	**22.0**	**1.4**	**8.7**	**18.9**	**19.3**	**21.0**	**17.4**	**17.4**	**21.3**	**37.5**	**20.7**	**1.4**	**9.5**	**19.6**	**20.5**	**24.3**	**18.6**	**18.6**	**20.6**	**27.8**	**24.0**	**1.7**	**9.5**	**18.7**	**20.0**	**20.4**	**18.3**	**18.3**		
Afternoon	181	7/19/23 14:32:19	19.2	27.1	21.4	0.8	8.0	19.4	18.3	20.3	16.3	16.3	20.3	37.4	20.9	0.4	8.7	22.5	19.5	24.2	17.5	17.5	21.5	33.8	23.2	0.5	9.9	22.8	20.9	23.7	19.2	19.2	Wet turf	Clear
	182	7/19/23 14:44:04	22.3	25.7	19.2	0.4	9.2	23.5	21.6	21.6	19.6	19.6	21.2	35.4	19.0	0.3	9.2	23.8	20.4	24.0	18.3	18.3	21.8	32.9	21.1	1.0	9.1	20.4	21.1	23.6	19.3	19.3	Wet turf	Clear
	183	7/19/23 14:53:02	21.5	27.3	20.1	0.5	9.3	22.2	20.8	21.7	18.8	18.8	21.7	35.1	19.3	0.7	9.3	21.1	20.9	24.2	18.9	18.9	21.7	34.5	21.0	1.1	9.7	19.9	21.0	23.9	19.2	19.2	Wet turf	Clear
	184	7/19/23 15:07:57	20.8	23.3	21.5	0.9	9.0	19.8	20.1	20.0	18.2	18.2	21.4	32.7	19.9	0.4	9.2	22.7	20.6	23.3	18.7	18.7	21.7	31.8	19.7	1.1	9.5	19.6	21.0	23.1	19.0	19.0	Wet turf	Clear
			**21.0**	**25.9**	**20.6**	**0.7**	**8.9**	**20.9**	**20.2**	**20.9**	**18.2**	**18.2**	**21.2**	**35.2**	**19.8**	**0.5**	**9.1**	**22.4**	**20.4**	**24.0**	**18.3**	**18.3**	**21.7**	**33.3**	**21.3**	**0.9**	**9.6**	**20.8**	**21.0**	**23.7**	**19.2**	**19.2**		
Afternoon	189	7/31/23 14:59:12	15.7	24.8	25.6	0.5	6.8	18.7	14.7	17.6	12.7	12.7	15.8	32.3	25.1	0.4	6.6	19.4	14.7	20.2	12.7	12.7	15.6	35.9	24.1	1.9	6.6	14.4	14.5	19.5	12.4	12.4	Dry turf	Clear
	190	7/31/23 15:06:01	14.9	21.1	23.3	0.5	5.5	17.8	13.7	15.9	11.5	11.5	15.1	30.2	24.7	1.0	5.9	15.8	14.0	19.1	11.9	11.9	14.9	29.6	23.9	1.0	5.3	15.6	13.7	18.8	11.6	11.6	Dry turf	Clear
	191	7/31/23 15:31:54	16.7	19.8	27.6	0.5	7.1	19.0	15.9	16.6	14.1	14.1	15.4	28.8	24.2	1.6	6.4	14.3	14.3	17.7	12.2	12.2	17.4	29.4	23.4	0.0	7.0	22.1	16.4	20.1	14.4	14.4	Dry turf	Clear
	192	7/31/23 15:48:10	15.8	15.5	27.7	1.5	6.6	14.8	14.9	13.7	13.0	13.0	15.7	25.1	26.3	0.6	6.7	17.2	14.7	17.8	12.7	12.7	15.1	24.3	25.2	1.5	6.5	14.1	14.0	16.2	11.9	11.9	Dry turf	Clear
			**15.8**	**20.3**	**26.1**	**0.8**	**6.5**	**16.9**	**14.8**	**16.2**	**12.8**	**12.8**	**15.5**	**29.1**	**25.1**	**0.9**	**6.4**	**16.2**	**14.4**	**19.0**	**12.4**	**12.4**	**15.8**	**29.8**	**24.2**	**1.1**	**6.4**	**15.7**	**14.6**	**19.2**	**12.6**	**12.6**		
		**Average**	**18.0**	**24.1**	**32.9**	**0.8**	**9.0**	**20.4**	**17.5**	**18.7**	**16.1**	**16.1**	**18.2**	**31.6**	**31.7**	**0.8**	**9.0**	**20.4**	**17.7**	**21.1**	**16.2**	**16.2**	**18.5**	**31.9**	**32.5**	**0.8**	**9.3**	**20.5**	**18.1**	**21.4**	**16.6**	**16.6**		
		**Maximum**	**22.3**	**32.7**	**53.7**	**2.6**	**11.5**	**26.3**	**21.6**	**22.9**	**20.4**	**20.4**	**22.5**	**38.2**	**52.6**	**3.9**	**11.4**	**26.7**	**22.3**	**25.1**	**21.1**	**21.1**	**21.8**	**46.4**	**54.0**	**3.3**	**12.1**	**26.5**	**21.2**	**27.4**	**20.1**	**20.1**		
		**Minimum**	**12.3**	**12.0**	**19.2**	**0.0**	**5.5**	**14.6**	**12.2**	**10.7**	**10.8**	**10.8**	**12.5**	**20.5**	**19.0**	**0.0**	**5.9**	**14.3**	**12.5**	**15.4**	**11.0**	**11.0**	**12.4**	**11.8**	**19.7**	**0.0**	**5.3**	**14.1**	**12.4**	**11.9**	**10.9**	**10.9**		

S6. Comparison of the UTCI calculated using values for radiative T_{surf} versus black bulb temperature values for the radiative heat variable.

03/12/2023, 12:00-14:00

Surface	UTCI with Radiant Heat Meter	UTCI with Black bulb Temperature	T-test
Grass	Average= 33.9<br>Standard Deviation = 0.7	Average = 32.3<br>Standard deviation = 1.6	T=1.803, p=0.1214<br>Difference not statistically significant
Court	Average = 40.3<br>Standard deviation = 1.2	Average = 33.2<br>Standard deviation = 1.6	T=7.1, p<0.0001<br>Difference is statistically significant
Turf	Average = 45.5<br>Standard deviation = 2.4	Average = 35.2<br>Standard Deviation = 2.2	T=6.3, p<0.0001<br>Difference is statistically significant
Average	Average = 39.9<br>Standard deviation = 5.2	Average = 33.6<br>Standard deviation = 2.1	T=3.9, p<0.0001<br>Difference is statistically significant

S7. Diagram showing *in situ* data collection protocol

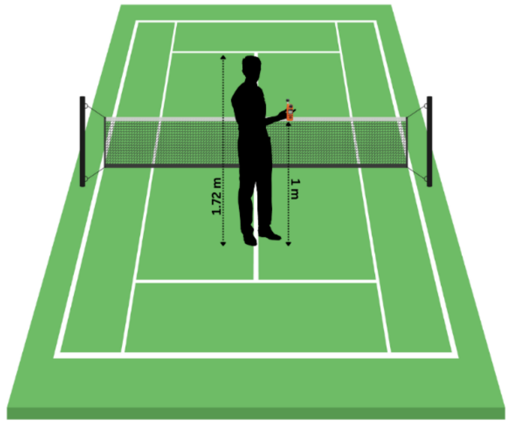

Supplement: Supplementary file 1 — Supplementary Material 1 [file 484_2024_2748_MOESM1_ESM.pdf]
